# Balancing Crystallization Kinetics and Lattice Strain Enables Thermally Stable Slot‐Die‐Coated Narrow‐Bandgap Perovskite Solar Cells

**DOI:** 10.1002/advs.78178

**Published:** 2026-10-06

**Authors:** Xiaojing Ci, Altantulga Buyan‐Arivjikh, Xiongzhuo Jiang, Simon A. Wegener, Christoph G. Lindenmeir, Lixing Li, Thomas Baier, Matthias Schwartzkopf, Sarathlal Koyiloth Vayalil, Kun Sun, Peter Müller‐Buschbaum

**Affiliations:** ^1^ TUM School of Natural Sciences Department of Physics Chair for Functional Materials Technical University of Munich Garching Germany; ^2^ Deutsches Elektronen‐Synchrotron DESY Hamburg Germany; ^3^ Applied Sciences Cluster University of Petroleum and Energy Studies UPES Dehradun Uttarakhand India; ^4^ Helmholtz‐Zentrum Berlin Für Materialien und Energie GmbH Berlin Germany

**Keywords:** growth kinetics, operando grazing‐incidence wide‐angle X‐ray scattering, seed crystals, Sn‐Pb perovskite, thermal cycling stability

## Abstract

Unbalanced crystallization kinetics in mixed Sn‐Pb perovskite cause film heterogeneity and high defect densities, thereby constraining overall performance and stability, particularly under scalable processing conditions. Here, we investigate nanocrystal‐mediated crystallization regulation in ambient‐processed, slot‐die‐coated narrow‐bandgap Sn‐Pb perovskites by combining CsPbI_3_ nanocrystal‐assisted processing with time‐resolved in situ grazing‐incidence wide‐angle X‐ray scattering (GIWAXS). After nine ISOS‐T‐3 thermal cycles, the perovskite solar cells (PSCs) fabricated from seeded perovskite films retain 80% of their initial efficiency at 25°C. *Operando* GIWAXS reveals differences in lattice evolution, residual PbI_2_ formation, and microstrain accumulation between the control and nanocrystal‐assisted films. By linking scalable processing and crystallization kinetics to the subsequent lattice response under thermal cycling, this work establishes a processing, crystallization, structure, stability relationship for narrow‐bandgap Sn‐Pb perovskites and highlights the importance of controlling early‐stage film formation for thermally robust, scalable perovskite photovoltaics.

## Graphical abstract

Unbalanced crystallization in mixed Sn‐Pb perovskites causes film defects and instability. CsPbI_3_ nanocrystals serve as nucleation seeds to regulate growth during slot‐die coating. Resulting solar cells retain 80% efficiency after nine thermal cycles. *Operando* GIWAXS confirms suppressed microstrain and lattice distortion, demonstrating that synergistic crystallization control and lattice regulation critically enhance thermal‐cycling stability.

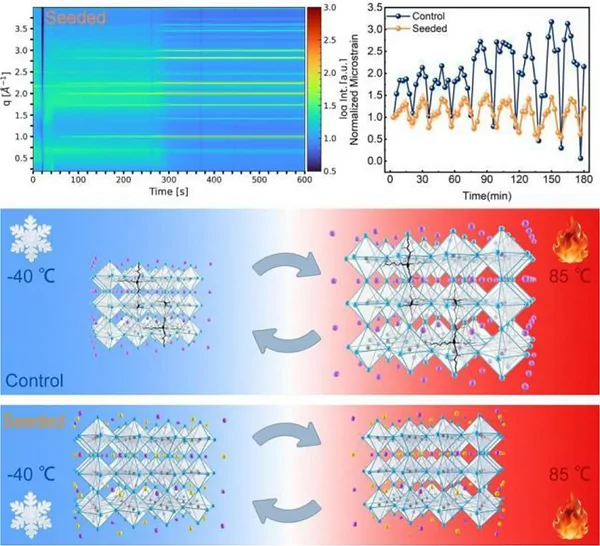

## 1 Introduction

Significant advances have been made in all perovskite tandem solar cells recently, which integrate a narrow‐bandgap bottom cell with a wide‐bandgap top cell. Tandem architectures enable a more efficient utilization of the solar spectrum [1, 2, 3, 4, 5]. Recently, certified PCEs of all‐perovskite tandem solar cells have exceeded 30%, demonstrating their considerable potential for high‐efficiency photovoltaic applications [6]. For narrow‐bandgap (NBG) PSCs designed for tandem applications, the effective bandgap tuning to an optimal range generally requires a partial substitution of lead (Pb) with tin (Sn), forming mixed Sn‐Pb perovskites. However, Sn^2+^ ions are highly susceptible to oxidation to Sn^4+^ under ambient conditions, leading to severe self‐p‐doping and the formation of deep defect states [7, 8, 9]. In addition, Sn‐based perovskites typically exhibit extremely fast crystallization kinetics, making it challenging to achieve uniform, dense films with controllable morphology over large areas [10, 11, 12]. Collectively, these intrinsic issues severely limit the scalable fabrication and long‐term operational stability of NBG Sn‐Pb mixed PSCs under ambient conditions, thereby hindering their practical implementation in commercial real‐world photovoltaic technologies.

Studies have demonstrated that perovskite nanocrystals (NCs) can be used as nucleation templates to provide lattice‐matched heterogeneous nucleation sites, thereby guiding the epitaxial or quasi‐epitaxial growth [13, 14, 15, 16]. This strategy significantly lowers the Gibbs free energy barrier for nucleation and markedly enhances both the nucleation rate and nucleation density [17, 18]. Experimental results further confirm that NC‐assisted nucleation not only facilitates the formation of highly oriented perovskite films with low defect densities but also substantially prolongs the lifetime of photogenerated charge carriers, leading to improvements in power conversion efficiency (PCE) in solar cells and other optoelectronic devices [19, 20]. In addition, a growing body of research indicates that A‐site cation engineering through the incorporation of inorganic cations with smaller ionic radii is an effective approach to enhancing the intrinsic stability of perovskite materials [21]. In particular, partial substitution of organic formamidinium (FA^+^) cations with inorganic cesium (Cs^+^) to construct mixed cation systems such as Cs_x_FA_1‐x_PbI_3_ has achieved significant advances in improving the phase stability and thermal stability of FAPbI_3_ perovskites. By optimizing the Goldschmidt tolerance factor, this strategy suppresses the formation of the non‐photoactive δ phase and increases lattice rigidity, thereby markedly enhancing structural robustness under ambient and thermal stress conditions. In addition, surface ligands of perovskite nanocrystals may also act as interfacial passivation agents, reducing defect‐assisted recombination and contributing to improved device performance [22, 23, 24, 25]. These improvements provide a critical materials foundation for the development of enhanced efficiency and perovskite photovoltaic devices. Although these studies demonstrate the effectiveness of nanocrystal‐assisted crystallization, A‐site cation regulation, and interfacial passivation for improving perovskite film quality and device performance, the dynamic structural mechanisms underlying these improvements remain insufficiently understood under scalable processing conditions. In particular, how nanocrystal‐assisted processing modifies the crystallization pathway of slot‐die‐coated narrow‐bandgap mixed Sn‐Pb perovskites, and how the resulting structural differences subsequently affect lattice evolution and irreversible degradation under repeated thermal cycling, have rarely been directly investigated. Establishing such a connection requires time‐resolved characterization that follows the material from its initial crystallization to its structural response under cyclic thermal stress.

In this study, we investigate the crystallization and thermal structural evolution of ambient‐processed narrow‐bandgap Sn‐Pb perovskites fabricated by two‐step slot‐die coating, using CsPbI_3_ nanocrystals to regulate the early‐stage film formation. Time‐resolved in situ GIWAXS is used to directly track the crystallization pathway during perovskite formation, allowing the dynamic structural evolution of the control and nanocrystal‐assisted films to be compared. The nanocrystal‐assisted process leads to earlier perovskite formation, modified subsequent phase evolution, and an absolute PCE enhancement of 1.55% compared with the control devices. More importantly, it results in improved thermal‐cycling stability relative to the control device. To further elucidate, in a systematic manner, the intrinsic correlation between efficiency degradation in NBG PSCs under thermal cycling stress and the associated crystal phase transitions and lattice structural evolution, *operando* GIWAXS measurements are performed in conjunction with temperature cycling tests conducted strictly in accordance with the ISOS‐T‐3 protocol (‐40°C to 85°C). The aging behavior of the PSCs can be distinctly divided into two characteristic stages. In the initial stage, the degradation is dominated by reversible elastic lattice deformation, which induces only minor modulation of charge transport properties. In the subsequent stage, the accumulation of thermal expansion mismatch leads to irreversible plastic deformation, resulting in the proliferation of lattice defects and a pronounced increase in the microstrain. These coupled effects severely degrade the optoelectronic performance of the devices, resulting in an irreversible PCE decay and enhanced periodic fluctuations of the solar cell parameters. This work demonstrates how scalable slot‐die processing can be combined with in situ and *operando* structural characterization to establish a direct relationship between crystallization kinetics, lattice evolution, and thermal cycling stability in narrow‐bandgap Sn‐Pb perovskites. It elucidates that balancing the growth kinetics and relieving the accumulated microstrain in NBG perovskites during thermal cycling are critical to enhancing the stability of perovskite solar cells printed under ambient conditions.

## 2 Results and Discussion

### 2.1 Perovskite Film Preparation via Slot‐Die Coating

Figure 1a illustrates the fabrication workflow of the printed perovskite thin films via two‐step slot‐die coating based on a seed‐assisted NCs strategy. In this process, pre‐synthesized CsPbI3 NCs are introduced as heterogeneous nucleation centers to regulate the perovskite crystallization. The photoluminescence properties of CsPbI_3_ NCs and their corresponding PL spectra are presented in Figures S1 and S2, respectively. Dynamic light scattering (DLS) measurements show that the CsPbI_3_ nanocrystals exhibit a characteristic hydrodynamic size of approximately 8 nm (Figure S1b). Oleylamine was used as the surface ligand of the CsPbI_3_ nanocrystals, and its molecular structure is shown in Figure S1c. As the first step of the two‐step perovskite fabrication process, a mixed tin‐lead iodide precursor solution is slot‐die coated onto a heated substrate (70°C), with nitrogen gas quenching to accelerate solvent evaporation and induce preliminary crystallization. Subsequently, for the seeded perovskite films, the NC film is slot‐die coated under nitrogen gas quenching conditions, leading to the formation of NC arrays. The NCs at this stage serve as seeds for the subsequent growth of perovskite crystals. Finally, the formamidinium iodide solution is further slot‐die coated on top of the mixed tin‐lead iodide films with and without NC seeds, denoted as the control and seeded samples, respectively.

**FIGURE 1:**
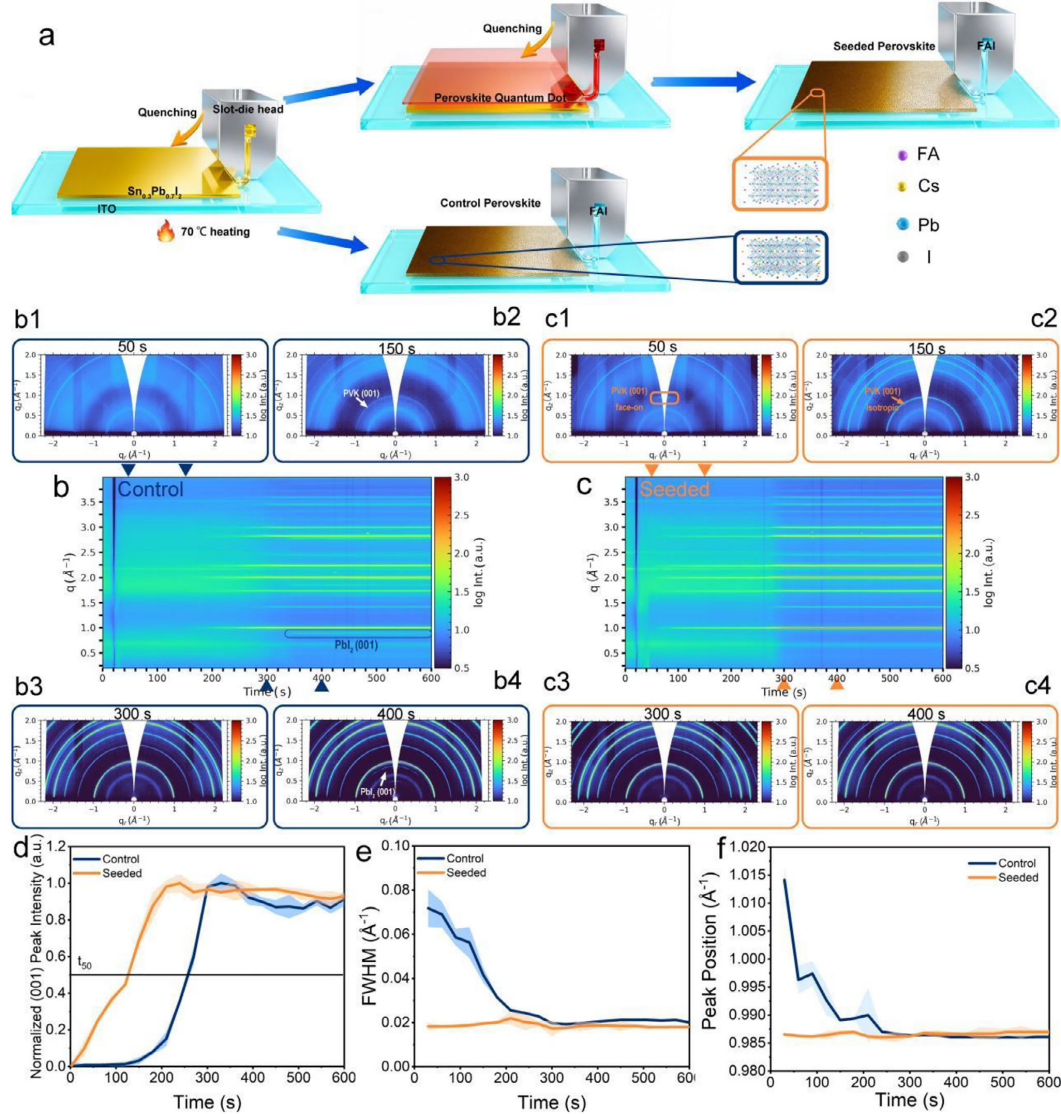
(a) Schematic diagram of the fabrication processes of slot‐die coated perovskite films. (b) Mapping of azimuthal cake cuts of the 2D GIWAXS data for the fabrication process of the control sample. Selected reshaped 2D GIWAXS data during the fabrication of the control sample at 50 s (b1), 150 s (b2), 300 s (b3), and 400 s (b4). (c) Mapping of azimuthal cake cuts for the fabrication process of the seeded sample. Selected reshaped 2D GIWAXS data during the fabrication of the seeded sample at 50 s (c1), 150 s (c2), 300 s (c3), and 400 s (c4). Temporal evolution of (d) normalized (001) peak intensity, (e) full width at half maximum of (001) peak, and (f) (001) peak position during crystallization.

To unravel in depth the role of NCs in assisting the nucleation and growth of perovskite thin films, in situ GIWAXS is used to monitor the crystallization kinetics and microstructural evolution of the printed perovskite films (Figure S3). The slot‐die coating process is carried out using a custom‐built setup (Figure S3b) [18]. Figure 1b,c present mappings of the temporal evolution of radially integrated cake cut intensities during the fabrication process via slot‐die coating for the control sample and the seeded sample, respectively. It is clearly observed that, for the control sample, a pronounced diffraction peak corresponding to the perovskite (001) crystal plane emerges after only approximately 150 s (as shown in Figure 1b1,b2). In contrast, for the seeded sample, a distinct (001) diffraction signal is detectable at around 50 s already (as shown in Figure 1c1,c2). This substantial advancement in the onset time of perovskite crystallization indicates that the NCs effectively accelerate the nucleation and growth kinetics of the perovskite [18, 26, 27]. In addition, as shown in Figure 1c1, the seeded sample exhibits a pronounced face‐on orientation of the (001) crystal planes during the early‐stage of perovskite crystallization. As the crystallization process proceeds to approximately 150 s, this preferential orientation gradually evolves into an isotropic distribution, as shown in Figure 1c2. To further investigate the evolution of crystal orientation during perovskite film formation, we look into the mapping of the azimuthal cuts along the {100} diffraction peak as seen in Figure S4. The control sample (Figure S4a) shows no pronounced evolution in crystal orientation, indicating kinetically dominated crystallization [28]. In contrast, seed‐induced epitaxial growth initially promotes an ordered orientation that gradually relaxes toward an isotropic distribution during film formation, relieving internal stress and improving thermal stability [29, 30].

As shown in Figure 1d–f, the crystallization kinetics and structural evolution of the control and seeded films are quantitatively evaluated from the normalized (001) diffraction intensity, FWHM, and peak position q_(001)_. The crystallization progress is defined as $X(t)=\frac{I(t)-I_{0}}{I_{\max}-I_{0}}$ where I_0_ and I_max_ are the initial and maximum (001) diffraction intensities, respectively [31]. The characteristic crystallization time *t_50_*, corresponding to X = 0.5, is approximately 117.6 s for the seeded film and 257.4 s for the control film, indicating an advancement of the crystallization process by approximately 140 s upon NCs seeding. The maximum apparent crystallization rate, $R(t)=\frac{\mathrm{dX}(t)}{\mathrm{dt}}$ [32], is approximately 0.0062 s^−1^ for the seeded film and 0.0126 s^−1^ for the control film. Thus, seeding primarily shifts crystallization toward earlier times rather than increasing the maximum crystallization rate, resulting in a more gradual crystallization pathway compared with the delayed rapid crystallization observed for the control film.

The FWHM evolution in Figure 1e further demonstrates the accelerated establishment of crystalline coherence. At 30 s, the seeded film exhibits a FWHM of only 0.02 Å^−1^, substantially smaller than that of the control film (0.08 Å^−1^). Using a Scherrer‐type relation, $L\approx \frac{2\pi K}{\Delta q}$ with K = 0.9 [33], the corresponding apparent coherent domain sizes are approximately 31.0 and 7.9 nm, respectively. At 330 s, the values converge to approximately 32.2 nm for the seeded film and 29.3 nm for the control film, indicating that the seeded film reaches a near final coherence length at a substantially earlier stage. Consistently, Figure 1f shows that the seeded film reaches q_(001)_≈0.98 Å^−1^ at 30 s and subsequently remains close to its final value of 0.99 Å^−1^. In contrast, the control film exhibits a pronounced shift from approximately 1.01 to 0.99 Å^−1^ during crystallization. The pronounced q position evolution in the control film further suggests substantial lattice and compositional rearrangement during the early crystallization stage. Previous studies have shown that the Sn‐containing component of mixed Sn‐Pb perovskites crystallizes more rapidly than its Pb counterpart, which can result in asynchronous cation incorporation and transient Sn‐enriched regions [34]. These results demonstrate that NCs seeding promotes earlier crystallization, rapid establishment of crystalline coherence, and earlier stabilization of the perovskite lattice.

At 240 s, all samples are subsequently subjected to thermal annealing at 150°C, and related selected reshaped 2D GIWAXS data for the control and seeded perovskite films are shown in Figure 1b3,b4,c3,c4, respectively. Notably, at approximately 400 s, a pronounced diffraction peak corresponding to PbI_2_ (001) emerges in the case of the control sample, whereas no such impurity phase signal is detected in the seeded sample. These results indicate that this template‐assisted growth ensures a synergistic incorporation of Sn and Pb cations, effectively bypassing the formation of the PbI_2_ secondary phase and achieving a more balanced crystallization pathway. Figures S5a,b show the pseudo XRD patterns of the control and seeded samples, respectively. It is observed that, compared with the control perovskite film, the seeded film exhibits markedly higher diffraction intensities at the characteristic diffraction peaks, indicating that the incorporation of NCs as nucleation centers effectively promotes crystal growth. Furthermore, to determine whether Cs introduced by the CsPbI_3_ nanocrystals is incorporated into the final perovskite film, XPS measurements are performed on both the control and seeded films, with particular attention to the high‐resolution Cs 3d spectra. As shown in Figure S6, a small Cs signal is detected in the seeded film, corresponding to an atomic concentration of approximately 0.5 at.%, whereas no detectable Cs signal is observed in the control film. These results confirm that the introduction of CsPbI_3_ nanocrystals leads to a small but measurable amount of Cs incorporation into the final perovskite film, resulting in a compositional difference between the seeded and control films.

The surface topography and microstructure of the control and seeded perovskite films are examined using atomic force microscopy (AFM) and scanning electron microscopy (SEM), as shown in Figures S7 and S8. The seeded perovskite thin film exhibits a reduced root‐mean‐square (RMS) roughness of 34.4 nm compared with 37.0 nm for the control film. Statistical analysis of 50 randomly selected grains from the AFM images in combination with fits using Gaussian distributions reveals that the control perovskite films possess an average grain size of approximately 0.97 µm, whereas the seeded perovskite films display a substantially larger average grain size of about 1.68 µm. Furthermore, SEM images indicate that the control perovskite films contain a higher density of pinholes and voids than the seeded counterparts. These observations collectively demonstrate that the introduction of nanocrystals markedly improves the surface morphology of perovskite thin films, resulting in smoother, more compact, and structurally uniform layers. As shown in Figure S9, the CsI‐only perovskite film exhibits a surface roughness of approximately 37.4 nm. Statistical analysis of 50 individual grains, followed by Gaussian fits of the grain‐size distribution, yields an average grain size of approximately 0.99 µm, which is comparable to that of the control film. No pronounced grain enlargement or morphological improvement is observed upon the addition of CsI. These results suggest that introducing a small amount of Cs into the perovskite composition alone has only a limited influence on the film morphology and cannot reproduce the morphological changes induced by NCs seeding. UV‐Vis absorption spectroscopy is used to investigate the optical properties of perovskite thin films. As shown in Figure S10, the control film exhibits an optical bandgap of 1.32 eV, whereas both the seeded and CsI‐only films show slightly widened bandgaps of approximately 1.35 eV, as extracted from the Tauc plots. Steady state PL spectra further reveal distinct photophysical differences between the control and seeded films (Figure S11). The seeded film exhibits enhanced PL intensity together with a blue shift of the emission peak from 943.5 to 929.5 nm. The enhanced PL intensity is consistent with improved optoelectronic quality.

To evaluate the depth‐dependent Sn/Pb distribution, high‐resolution XPS measurements are performed on the film surface and after 200 s of Ar ion etching, as shown in Figure S12. The control film shows a pronounced variation in the Pb/Sn atomic ratio, decreasing from 3.9 at the surface to 1.9 in the deeper region, indicating a substantial vertical compositional gradient. In contrast, the seeded film exhibits much more consistent Pb/Sn ratios of 3.6 and 3.4, respectively, supporting a substantially reduced depth‐dependent Sn/Pb compositional gradient in the seeded film. This suggests that NC‐mediated nucleation promotes more spatially uniform precursor conversion and reduces local compositional imbalance. In addition, stronger oxidized Sn and Pb‐related components are observed in the control film, whereas these species are markedly suppressed in the seeded film. In particular, the reduced Sn^4+^ contribution is consistent with suppressed oxidation of Sn^2+^, supporting a more homogeneous and chemically stable crystallization process in the seeded films.

### 2.2 Photovoltaic Performance

To investigate the effect of seed incorporation on the photovoltaic performance, PSCs with a configuration of indium tin oxide (ITO)/poly(3,4‐ethylenedioxythiophene) polystyrene sulfonate (PEDOT:PSS)/perovskite/[6,6]‐phenyl C_61_ butyric acid methyl ester (PC_61_BM)/bathocuproine (BCP)/Ag are fabricated from printed control and seeded perovskite films and systematically compared (Figure 2a). The current density voltage (J‐V) characteristics of the champion PSCs fabricated from printed control and seeded perovskite films are shown in Figure 2b,c, respectively, and the corresponding photovoltaic parameters extracted from the J‐V curves are summarized in the inset table. As expected, the seeded perovskite device displays a superior PCE of 18.06%, with an open circuit voltage (*V_OC_*) of 0.81 V, a short circuit current density (*J_SC_*) of 29.03 mA cm^−2^, and a fill factor (FF) of 76.77% under reverse scan conditions, along with a low hysteresis index of 1.24%. In contrast, the control device exhibits a PCE of 16.51%, with a *V_OC_* of 0.78 V, a *J_SC_* of 28.07 mA cm^−2^, an FF of 75.39%, and a slightly higher hysteresis index of 1.68%. To further elucidate the impact of NC seeds on the device performance, the statistical distributions of PCE and the associated photovoltaic parameters for PSCs fabricated from printed control and seeded perovskite films are presented in Figure 2d–g. The average PCE of the seeded perovskite devices increases from 15.91 ± 0.04% to 17.33 ± 0.07%, which arises from the collective enhancement of *V_OC_* (from 0.770 ± 0.001 V to 0.790 ± 0.002 V), *J_SC_* (from 27.27 ± 0.06 mA cm^−2^ to 28.38 ± 0.03 mA cm^−2^), and FF (from 74.63 ± 0.05% to 76.15 ± 0.06%). To evaluate whether the improvement in photovoltaic performance is related to the compositional change of the perovskite film, CsI‐only perovskite solar cells are fabricated as an additional control. The champion CsI‐only device (Figure S13a) achieves a PCE of 16.73%, with a *J_SC_* of 28.09 mA cm^−2^, a *V_OC_* of 0.79 V, and an FF of 75.37%. Statistical analysis of 20 independent devices (Figure S13b) yields average values of 27.43 ± 0.34 mA cm^−2^ for *J_SC_*, 0.772 ± 0.011 V for *V_OC_*, 74.75 ± 0.30% for FF, and 15.91 ± 0.38% for PCE. These photovoltaic parameters are generally comparable to those of the control devices and do not reproduce the pronounced performance enhancement observed for the seeded devices. These results indicate that the minor compositional modification induced by Cs incorporation has only a negligible influence on the photovoltaic performance under the conditions investigated here. Considering the corresponding optical bandgaps, the *V*_*OC*_ deficits of the champion control, seeded, and CsI‐only devices are approximately 0.54, 0.54, and 0.56 V, respectively. Thus, the 30 mV increase in *V*_*O*C_ from the control to the seeded device is essentially accounted for by the approximately 30 meV bandgap widening and should not be interpreted as evidence of reduced non‐radiative voltage loss. The photovoltaic benefit of NC seeding is therefore mainly reflected in the increased J_SC_ and FF.

**FIGURE 2:**
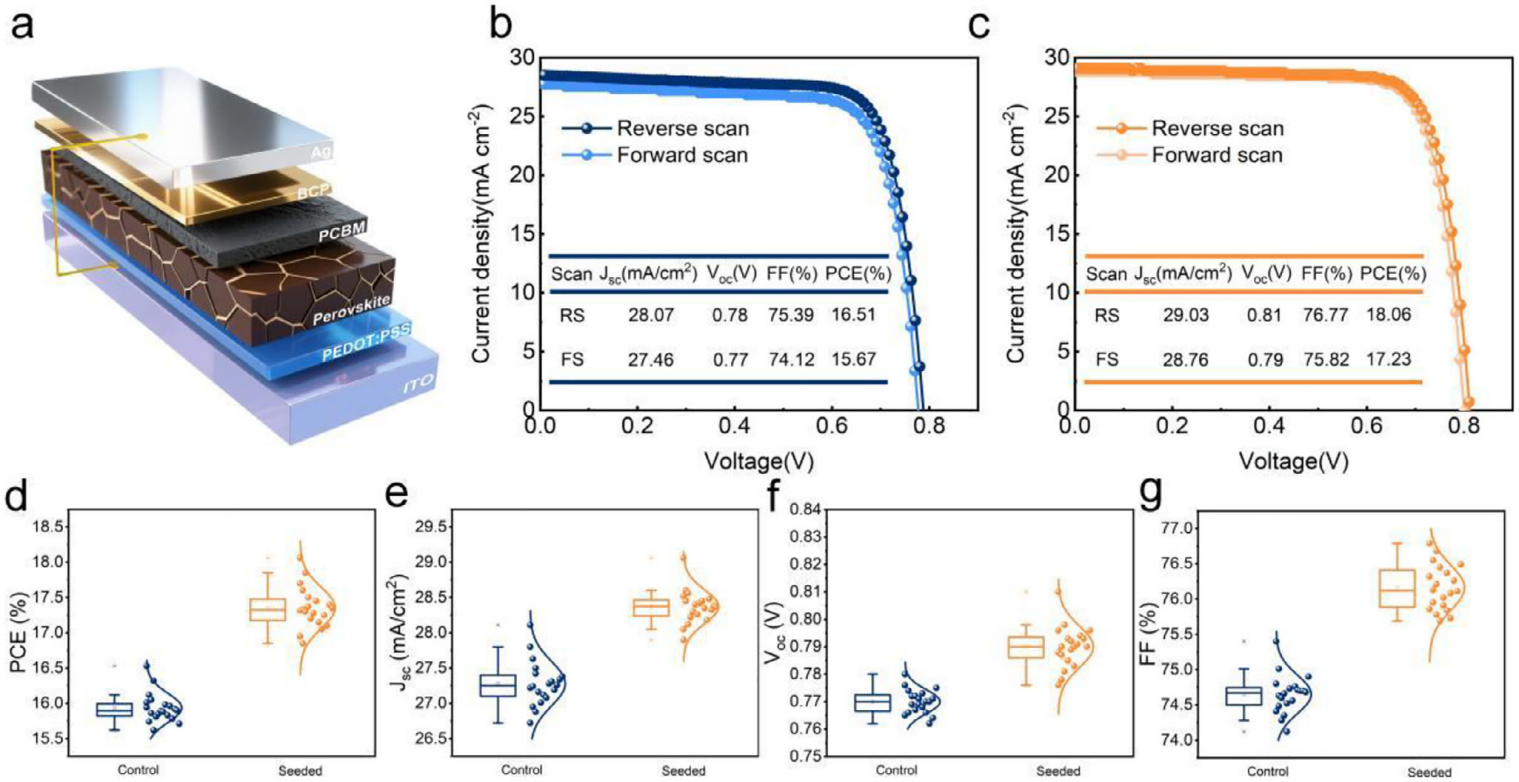
(a) Device layout of the PSCs. J‐V curves with forward and reverse scans of the champion PSCs fabricated from printed (b) control and (c) seeded perovskite films. Box plots of the distribution of (d) PCE, (e) *J_SC_*, (f) *V_OC_*, and (g) FF values of the PSCs fabricated from printed control (blue) and seeded (orange) perovskite films (20 devices for each type).

The observed enhancement in PCE of the seeded device can be primarily attributed to reduced recombination related losses, as evidenced by the high recombination resistance (*R_rec_*, Figure S14) and low dark current (Figure S15). Figure S14 presents the Nyquist plots obtained from EIS measurements for both control and seeded PSCs. In each case, a dominant semicircle appears in the high frequency region (10^3^–10^6^ Hz), which is mainly associated with the recombination resistance (*R_rec_*) in parallel with the device capacitance. Quantitative analysis yields *R_rec_* values of 653 and 1315 Ω cm^2^ for the control and seeded devices, respectively. Given that the recombination rate is inversely proportional to *R_rec_*, the substantially higher *R_rec_* of the seeded devices indicates a pronounced reduction in charge carrier recombination. These results suggest that the surface ligands of the NCs can effectively passivate surface and grain‐boundary defects in the perovskite thin films, thereby effectively suppressing nonradiative recombination within both the films and the corresponding devices [35]. The observed enhancement in the PCE of the seeded device can be primarily attributed to the reduced recombination related losses, as evidenced by the high *R_rec_* and low dark current (Figure S15). Collectively, these findings confirm that NCs as heterogeneous nucleation templates play a critical role in mitigating nonradiative recombination and enhancing overall device performance [36].

The EQE spectra of the control and seeded devices are shown in Figure S16, yielding integrated *J_SC_* values of 26.63 and 27.58 mA cm^−2^, respectively, consistent with the trend observed from the J‐V measurements. A clear blue shift of the absorption edge is observed in the seeded device, confirming a slight bandgap widening upon CsPbI_3_ introduction. Despite this spectral narrowing, the seeded device delivers a higher integrated photocurrent, indicating that the J_SC_ enhancement primarily arises from improved photogenerated carrier collection rather than from extended optical absorption. This improvement is attributed to the reduced recombination and improved film quality induced by nanocrystal‐mediated crystallization. To place the present results in the context of scalable NBG Sn‐Pb photovoltaics, recently reported device performance and stability are summarized in Table S1.

### 2.3 Operational Stability Under Thermal Cycling

To evaluate the thermal and operational stability of NBG PSCs under thermal cycling conditions, PSCs fabricated from printed control and seeded perovskite films are subjected to accelerated thermal cycling aging tests (ISOS‐T‐3 protocol) for 9 thermal cycles, over a temperature range from −40°C to 85°C [37]. Device performance was monitored at 3‐minute intervals, with each thermal cycle lasting 20 min. Analysis of the temporal evolution of the key photovoltaic parameters reveals that, although both device types exhibit similar degradation trends, the extent of performance deterioration differs markedly. Specifically, the control device (Figure 3a) retains only 58% of its initial PCE after 9 thermal cycles. This pronounced degradation is primarily driven by a concurrent decline in *J_SC_*, *V_OC_*, and FF, which fell to 78%, 88%, and 85% of the initial value, respectively. In contrast, the seeded device (Figure 3b) demonstrates substantially improved thermal cycling stability, maintaining 80% of its initial PCE, with *J_SC_*, *V_OC_*, and FF preserved at 90%, 95%, and 93% of their respective initial values. These pronounced differences indicate that incorporating NCs as nucleation seeds effectively enhances the structural and interfacial stability of the PSCs under thermal cycling. Furthermore, the coupled effects of electric field bias and periodic temperature variation may jointly contribute to the degradation kinetics observed under thermal cycling conditions, which differ substantially from those encountered under isothermal, static aging environments [38].

**FIGURE 3:**
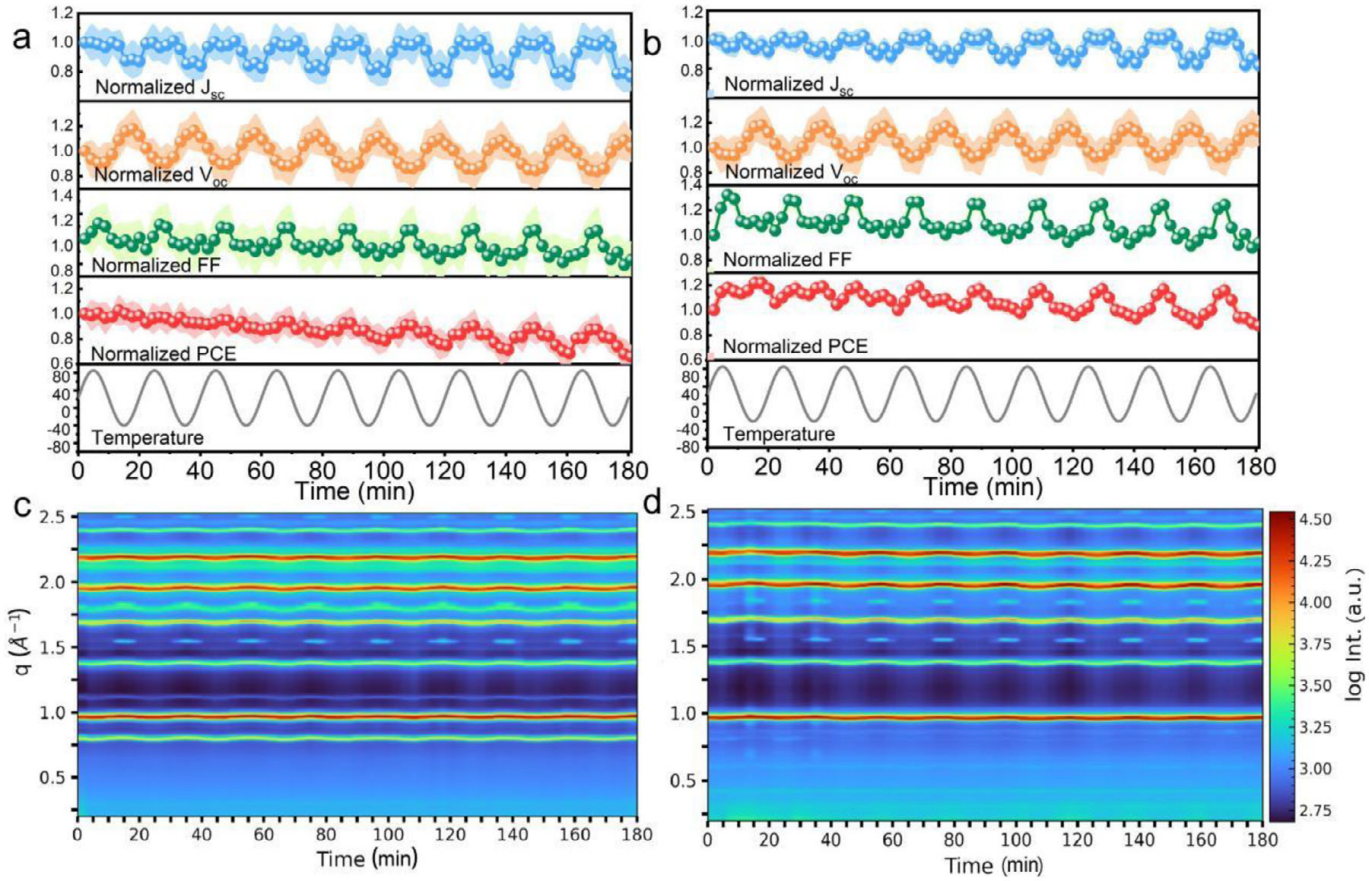
Evolution of the device performance under ISOS‐T‐3 conditions for PSCs fabricated from printed (a) control and (b) seeded perovskite films. The solid curves represent the mean values, and the shaded regions represent the error bars. Mapping of the pseudo XRD data extracted from the 2D GIWAXS data of PSCs fabricated from printed (c) control and (d) seeded perovskite films during thermal cycling conditions.

In addition, it is noteworthy that, compared with the subsequent six thermal cycles, the control devices exhibit less PCE degradation during the first three cycles, and the corresponding variations lack pronounced periodicity. In contrast, during the following six cycles, the PCE decays dramatically and displays pronounced periodic fluctuations that are highly synchronized with the temperature cycling. This behavior can be attributed to a transition in the dominant degradation mechanisms at different stages of thermal cycling. During the initial cycles, the device performance is primarily governed by thermally induced modulation of the charge transport and the ion migration, which has only a minor impact on device efficiency and does not exhibit clear periodic characteristics [39, 40]. However, with further aging, the accumulated thermal stress arising from mismatches in the coefficients of thermal expansion evolves from elastic strain to likely irreversible plastic deformation. This stress accumulation couples with defect generation and instability within the perovskite lattice, thereby triggering an irreversible microstructural degradation. Such structural damage not only deteriorates the device performance but also amplifies the sensitivity of its electrical response to periodic temperature variations [41, 42]. As a result, the PCE exhibits more pronounced, temperature‐synchronized oscillations during the aging thermal cycles. These findings underscore the critical role of thermomechanical stability in determining the long‐term cyclic reliability of PSCs.

Notably, *J_SC_* and FF are the two most sensitive performance metrics to thermal cycling. In particular, the evolution of *J_SC_* during temperature cycling exhibits a complex, non‐monotonic behavior. During the heating stage, *J_SC_* generally remains relatively stable or shows a slight increase. This behavior can be attributed to bandgap narrowing induced by elevated temperatures, which results in a marginal increase in *J_SC_* [43, 44]. In contrast, *J_SC_* decreases markedly in the low temperature regime. This decline primarily arises from a temperature‐induced bandgap broadening. Although *J_SC_* may exhibit reversible fluctuations with temperature within an individual thermal cycle, its overall trend becomes irreversibly degradative upon repeated thermal cycling [45, 46]. FF exhibits a pronounced temperature dependence during thermal cycling. During the heating stage, the FF initially increases rapidly to a peak value followed by a sharp decline. This initial enhancement can be attributed to the thermally activated electrical conductivity of the charge‐blocking layers; as the temperature rises, the reduction in overall series resistance temporarily boosts the FF [47, 48]. In the remaining temperature regimes, such as under low temperature conditions or prolonged exposure to elevated temperatures, the FF shows an overall downward trend. This suggests that the benefits of enhanced conductivity are outweighed by the effects of increased recombination‐related losses and structural fatigue in the perovskite lattice [49].

The thermal cycling stability of the CsI‐only devices is further evaluated over nine ISOS‐T‐3 cycles, as shown in Figure S18. After thermal cycling, the *J_SC_*, *V_OC_*, FF, and PCE retain approximately 81%, 90%, 89%, and 65% of their initial values, respectively. Compared with the control devices, incorporating a small amount of Cs yields a modest improvement in thermal cycling stability, suggesting that Cs alloying and the associated lattice stabilization may provide an additional beneficial contribution to device stability. Nevertheless, the improvement in stability remains substantially smaller than that observed for the seeded devices, indicating that Cs incorporation alone cannot account for the pronounced enhancement in thermal cycling stability achieved by NC seeding.

### 2.4 Structural Evolution Under Thermal Cycling

To establish a direct correlation between microstructural evolution and macroscopic device performance degradation of NBG PSCs under ISOS‐T‐3 conditions, *operando* GIWAXS measurements are performed, as illustrated in Figure S17. The GIWAXS results of PSCs fabricated from printed control and seeded perovskite films reveal that the characteristic perovskite diffraction peaks exhibit pronounced periodic oscillations with temperature cycling (Figure 3c,d). This reversible modulation of the characteristic Bragg peak position directly reflects periodic thermal expansion and contraction of the lattice parameters, indicating that the perovskite lattice undergoes significant thermally induced distortion within the applied temperature range. In addition, low temperature‐induced reversible phase transitions are observed in both control and seeded PSCs. Upon cooling, a fraction of the lattice transforms from the cubic (α) phase to the tetragonal (β) phase, giving rise to characteristic diffraction features at q ≈ 1.65 and 1.70 Å^−^^1^ (Figure S19) [50]. This phase transition is fully reversible upon reheating, suggesting that it originates from a thermodynamically driven phase transition rather than irreversible structural degradation.

To quantitatively evaluate the lattice response during thermal cycling, the relative lattice strain is calculated from the temperature‐dependent shift of the perovskite diffraction peak at q≈ 1.4 Å^−1^, using $\varepsilon \approx -\frac{q-q_{0}}{q_{0}}$, where q_0_ is the initial peak position at 25°C. As shown in Figure S21a, the control film exhibits a strain range of approximately −1.23% to +0.94%, corresponding to a peak‐to‐peak variation of 2.17%, whereas the seeded film is confined to ‐0.39% to +0.63%, giving a substantially smaller range of 1.02%. Furthermore, the q‐T trajectories over nine consecutive thermal cycles show markedly improved cycle‐to‐cycle reproducibility for the seeded film (Figure S21b,c). The average cycle‐to‐cycle standard deviation of the peak position decreases from 5.62 × 10^−3^ Å^−1^ for the control to 2.67 × 10^−3^ Å^−1^ for the seeded film, indicating a more reversible and stable thermoelastic lattice response.

To systematically elucidate the dynamic evolution of perovskite lattice parameters during thermal cycling, we track the evolution of the (002) diffraction planes of the control and seeded perovskite films, in both the out‐of‐plane and in‐plane directions. The results reveal that the control sample exhibits pronounced periodic shifts in the (002) q values. During the high temperature stages, the diffraction peaks shift toward lower q values, indicative of lattice expansion, whereas during the low temperature stages, they move toward higher q values, reflecting lattice contraction (Figure 4a,b). In contrast, although the seeded sample shows a similar temperature‐dependent trend, the overall magnitude of the q value remains essentially unchanged, indicating a greater resistance of the lattice structure to thermal perturbations (Figure 4c,d). After nine thermal cycles, the (002) interplanar spacing of the control sample increases by approximately 2%, whereas that of the seeded sample remains nearly unchanged, demonstrating its superior structural stability. The (001) crystal plane also yielded the same conclusion (as shown in Figure S20). This finding further demonstrates that the NC incorporation in the case of the seeded sample facilitates the formation of a highly symmetric and thermodynamically stable cubic phase and enhances the lattice robustness against external thermal disturbances, thereby suppressing large fluctuations in lattice parameters during thermal cycling [51].

**FIGURE 4:**
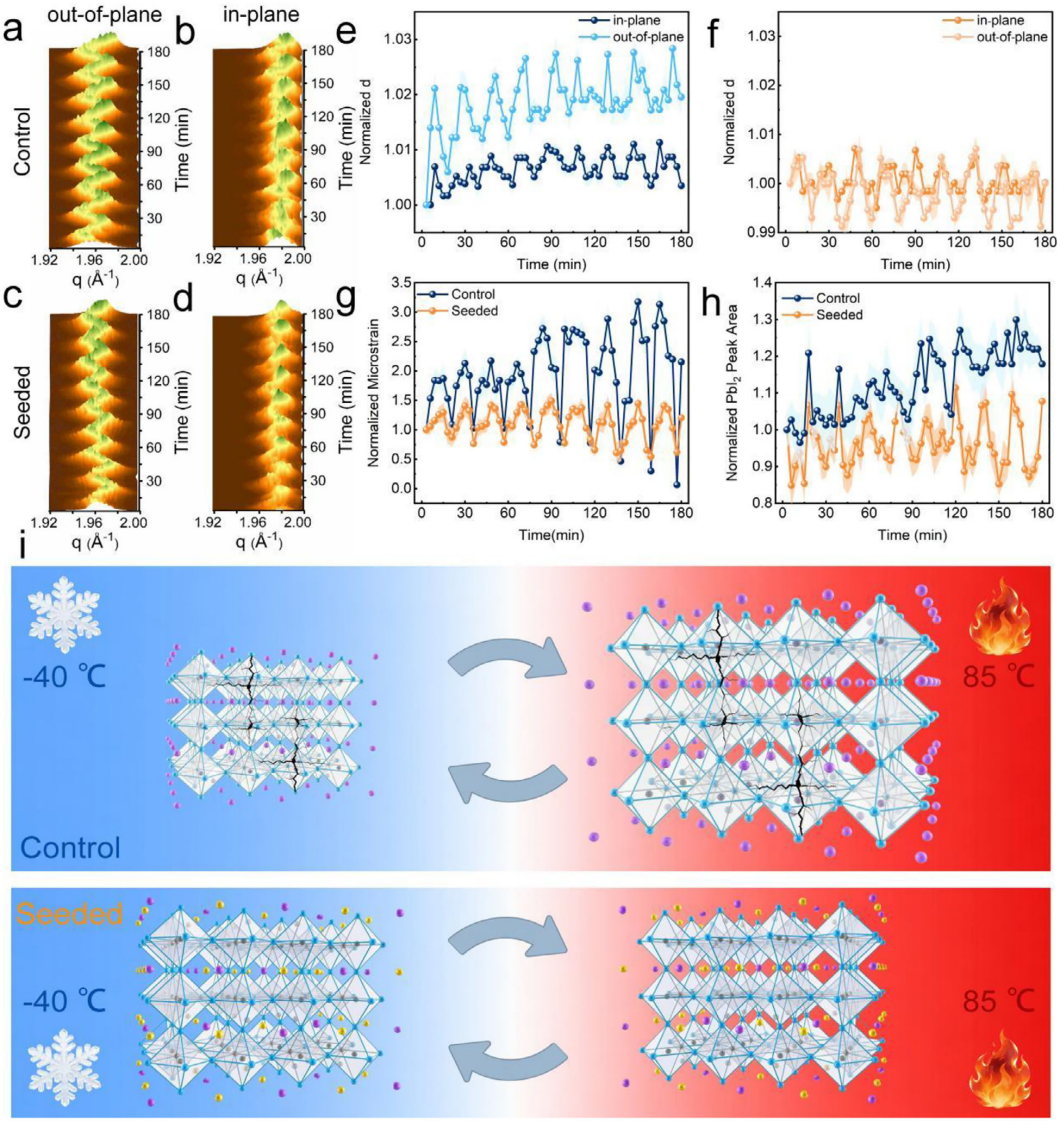
(a–d) 3D plot of time‐dependent variations in q positions for the (002) diffraction planes extracted from the operando GIWAXS data of PSCs fabricated from printed (a,b) control and (c,d) seeded perovskite films, in both the out‐of‐plane (a,c) and in‐plane (b,d) directions, with the z‐axis representing the intensity. Time‐dependent variations in normalized lattice spacing d for the (001) diffraction planes of the (e) control and (f) seeded PSCs. (g) Microstrain evolution calculated based on the Williamson‐Hall method for control and seeded PSCs. (h) Normalized integration of PbI_2_ peak area for control and seeded PSCs. (i) Schematic diagram of the lattice distortion under thermal cycling.

We also find anisotropic lattice distortion along the in‐plane and out‐of‐plane directions. This anisotropic behavior can be attributed to the intrinsically “soft” lattice nature of perovskite materials and the mismatch in thermal expansion between the perovskite layer and the adjacent functional layers [52]. Upon thermal cycling, the perovskite lattice experiences constrained out‐of‐plane expansion and contraction due to the lower‐CTE substrate, leading to alternating compressive and tensile stresses, while the in‐plane deformation is largely suppressed by a strong mechanical anchoring. Excessive stress accumulation induces microstrain and microcrack formation, thereby accelerating the device degradation. This mechanistic interpretation is validated by microstrain analysis of the *operando* GIWAXS data using the Williamson‐Hall method (Figure 4g) [53]. After 9 ISOS‐T‐3 thermal cycling, the microstrain in the control sample increases to approximately twice its initial value, whereas the seeded sample shows only a minor increase to about 1.3 times the initial value, indicating that the NC seeding strategy effectively mitigates thermal stress accumulation. Notably, during the first three thermal cycles, the microstrain in the control sample exhibits small and reversible fluctuations with temperature, reflecting a response dominated by elastic deformation. However, with increasing cycle number, the microstrain fluctuates significantly and cannot fully recover, closely correlating with the irreversible decay in device performance. This observation further confirms that, in the later stages of thermal cycling, strain induced by thermal expansion mismatch progressively evolves from reversible elastic deformation into irreversible plastic deformation, coupling with defect proliferation. Ultimately, these processes lead to irreversible structural degradation of the perovskite film, thereby severely deteriorating the photovoltaic performance and long‐term operational stability of the devices.

As shown in Figure 4h, quantitative analysis of the integrated intensity of the PbI_2_ diffraction peak allows tracking the temporal evolution of the degradation byproducts during thermal cycling. Although the integrated peak areas of PbI_2_ increase continuously in both types of devices, devices based on seeded perovskites exhibit the smallest overall increase throughout the aging period. In contrast, the control perovskite devices show the most pronounced PbI_2_ formation, consistent with their inferior device stability. This behavior can be attributed to irreversible chemical degradation induced by thermal cycling. Notably, the alloying of the NC seeds significantly enhances the thermochemical stability of the perovskite material, effectively increasing lattice rigidity and suppressing thermally induced phase segregation and decomposition reactions [54].

In summary, PSCs built from perovskite thin films fabricated with NCs as nucleation seeds exhibit markedly enhanced structural stability under thermal cycling. The temperature‐induced variations in lattice parameters are substantially suppressed, effectively mitigating microstrain accumulation and resulting in reduced lattice distortion and superior thermal cycling stability, as illustrated in Figure 4i. This enhanced stability can be attributed to the formation of highly crystalline, highly symmetrical cubic phase perovskite structures induced by NCs seeding, together with the regulatory effect on lattice thermal expansion behavior.

## 3 Conclusion

This study establishes a direct relationship between crystallization kinetics, lattice evolution, and thermal stability in scalable slot‐die‐coated NBG Sn‐Pb perovskite solar cells. By introducing CsPbI_3_ nanocrystals during film formation and combining the scalable coating process with time‐resolved in situ GIWAXS, we directly reveal substantial differences in the crystallization pathway between the control and nanocrystal‐assisted perovskite films, including earlier perovskite formation and suppressed residual PbI_2_ formation. These modifications in film formation are accompanied by improved film quality, reduced recombination‐related losses, and enhanced photovoltaic performance. As a result, the seeded PSCs exhibit reduced recombination‐related losses, along with enhanced photovoltaic performance and improved stability under ISOS‐T‐3 conditions. After nine thermal cycles, it retains 80% of its initial PCE at 25°C, compared with 58% for the control device. By using *operando* GIWAXS measurements, we systematically elucidate the intrinsic correlations between device aging behavior and the evolution of the crystal structure and phase transitions under ISOS‐T‐3 (−40°C to 85°C) conditions. The degradation is initially governed by reversible elastic lattice deformation. However, with continued aging, the accumulated thermal stress arising from mismatches in the coefficients of thermal expansion progressively evolves from elastic strain into irreversible plastic deformation, ultimately triggering permanent structural degradation and temperature‐synchronized variations in PCE. Overall, this work not only clarifies the structural degradation of NBG PSCs under cyclic thermal stress but also establishes a mechanistic connection between the initial crystallization pathway, subsequent lattice response, and device degradation. These findings highlight crystallization and lattice‐stability engineering as important strategies for improving the thermal robustness of scalable NBG perovskite photovoltaics.

## 4 Experimental Section

### 4.1 Materials

Lead iodide (PbI_2_, 99.999%), tin iodide (SnI_2_, 99.99%), formamidinium iodide (FAI, 98%), methylammonium iodide (MAI, 98%), methylammonium chloride (MACl), [6,6]‐phenyl C_61_ butyric acid methyl ester (PC_61_BM, 99.5%), bathocuproine (BCP, 99%), N,N‐anhydrous dimethylformamide (DMF, 99.8%), dimethyl sulfoxide (DMSO, 99.9%), isopropanol (IPA, 99.9%), chlorobenzene (CB, 99.8%) were purchased from Sigma–Aldrich. Poly(3,4‐ethylenedioxythiophene) polystyrene sulfonate (PEDOT:PSS, BaytronPVPAI4083) was purchased from Ossila. All materials were used directly without further purification.

### 4.2 Perovskite Film Printing and Device Preparation

The indium tin oxide (ITO) coated glass substrates were sequentially cleaned by ultrasonication in diluted Hellmanex III solution (2:98, v/v), deionized water, acetone, 2‐propanol, and ethanol, with each step lasting 15 min. The substrates were subsequently dried under a nitrogen gas stream. Prior to device fabrication, the ITO substrates were treated with oxygen plasma for 15 min. A PEDOT:PSS hole‐transport layer was deposited onto the ITO substrates by spin‐coating at 4000 rpm for 30 s, followed by thermal annealing at 120°C for 15 min. The perovskite absorber layer was fabricated in ambient air via a two‐step slot‐die coating process. In the first step, a precursor solution containing 1.05 M PbI_2_ and 0.45 M SnI_2_ dissolved in a DMF:DMSO mixed solvent (4:1, v/v) was coated onto the PEDOT:PSS/ITO substrates at 70°C using a home‐built slot‐die coater, with a printing speed of 20 mm s^−1^, a gap height of 200 µm, and a pump rate of 200 µL min^−1^. In the second step, a CsPbI_3_ NCs dispersion (5 mg mL^−1^) was slot‐die coated at 25°C with a printing speed of 10 mm s^−1^, maintaining the same gap height and pump rate. Subsequently, a solution of FAI:MACl:MAI (90:9:6 mg dissolved in 1 mL isopropanol) was coated at 25°C with a printing speed of 5 mm s^−1^ (gap height 200 µm, pump rate 200 µL min^−1^), followed by thermal annealing at 150°C for 15 min in ambient air. For electron transport layer deposition, a PC_61_BM solution (20 mg mL^−1^ in chlorobenzene) was dynamically spin‐coated onto the ITO/PEDOT:PSS/perovskite stack at 2500 rpm for 30 s. A BCP interlayer (0.5 mg mL^−^^1^ in isopropanol) was then dynamically spin‐coated at 5000 rpm for 30 s. Finally, a 100 nm thick silver electrode was thermally evaporated through a shadow mask. The active area of each device pixel was approximately 0.2 cm^2^.

### 4.3 GIWAXS Measurement

Grazing‐incidence wide‐angle X‐ray scattering (GIWAXS) measurements were performed at the P03/MiNaXS beamline of the PETRA III storage ring at DESY. Data acquisition was conducted using a LAMBDA 9 M detector. The X‐ray beam had a photon energy of 11.79 keV, corresponding to a wavelength of 1.044 Å, and the sample‐to‐detector distance (SDD) was set to 209.5 mm in slot‐die coating measurements and 250 mm in *operando* GIWAXS measurements. An incident angle (α_i_) of 0.6° was employed during slot‐die coating and *operando* GIWAXS measurements to capture scattering from the ITO substrate, which served as a calibration reference. Image processing was carried out using the Python‐based INSIGHT software, including transformations to q‐space, geometric corrections, and intensity adjustments accounting for path‐length attenuation, detector absorption, photon polarization, and solid‐angle effects [55]. Due to thermal expansion under illumination, the SDD was corrected using the ITO substrate signal at q = 2.139 Å^−1^. Radial cake cuts for pseudo XRD analysis and azimuthal tube cuts for texture/orientation analysis were performed with INSIGHT. Radial cuts were defined with χ = −90° to 90° and q = 0 to 2.5 Å^−1^, whereas azimuthal cuts for texture evaluation used χ = ‐90° to 90°.

### 4.4 Characterization

Atomic force microscopy (AFM) measurements were performed under ambient conditions using a Nanosurf FlexAFM system. Cross‐sectional and top‐view scanning electron microscopy (SEM) images were obtained using Zeiss Gemini NVision 40 and ZEISS EVO instruments. Cross‐sectional samples were examined at an accelerating voltage of 5 keV with a working distance of 6 mm, whereas surface morphology was characterized at 30 keV and a 9 mm working distance. Optical absorption spectra were recorded with a PerkinElmer Lambda 35 UV‐Vis spectrophotometer at a scan rate of 480 nm/min. Current density‐voltage (J‐V) characteristics were measured under simulated AM 1.5G illumination (100 mW cm^−2^) using a Newport Class ABA solar simulator coupled with a Keithley 2611B source meter, calibrated with a certified silicon reference cell (Fraunhofer ISE 019–2015). All devices were tested through an aperture mask with an active area of 0.079 cm^2^, employing a scan rate of 50 mV/s, a voltage step of 10 mV, and a dwell time of 100 ms per step.

### 4.5 Statistical Analysis

Photovoltaic parameters were statistically evaluated using 20 independent devices fabricated from different preparation batches for each condition. The data are presented as mean ± standard deviation. Grain size distributions were obtained by measuring 50 individual grains from the corresponding microscopy images. Except for the thermal‐cycling and GIWAXS quantities explicitly normalized as described in the text, no additional data transformation or outlier exclusion was applied. For the quantitative GIWAXS analysis, the shaded regions shown in the corresponding plots represent the fit uncertainties derived from the peak fit procedure. No additional statistical software was used for the statistical analysis.

## Author Contributions

X. Ci conceived the idea, designed the research, performed the experiments, analyzed the data, and wrote the manuscript. A. Buyan‐Arivjikh provided the nanocrystals. S.A. Wegener built the thermal cycling setup. A. Buyan‐Arivjikh, X. Jiang, S.A. Wegener, C.G. Lindenmeir, L. Li, T. Baier, and K. Sun analyzed the data and discussed the results. S.K. Vayalil and M. Schwartzkopf provided resources. P. Müller‐Buschbaum conceived the idea, provided funding, project administration, and supervised the research. All authors discussed the results, provided critical feedback, contributed to, and approved the final version of the manuscript.

## Conflicts of Interest

The authors declare no conflicts of interest.

## Supporting information




**Supporting File**: advs78178‐sup‐0001‐SuppMat.pdf.

## Data Availability

The data that support the findings of this study are openly available in mediatum at https://mediatum.ub.tum.de/1860573.
