# Supplementary material for: Balancing Crystallization Kinetics and Lattice Strain Enables Thermally Stable Slot‐Die‐Coated Narrow‐Bandgap Perovskite Solar Cells
Source: Adv Sci (Weinh). 2026 Oct 6:e78178. Online ahead of print. doi: 10.1002/advs.78178 (PMC13643118; doi:10.1002/advs.78178)
Supplement: Supplementary file 1 [file ADVS-9999-e78178-s001.pdf]

Supporting Information

Balancing Crystallization Kinetics and Lattice Strain Enables Thermally Stable Slot-Die-Coated Narrow-Bandgap Perovskite Solar Cells

Xiaojing Ci[a], Altantulga Buyan-Arivjikh[a], Xiongzhuo Jiang[a], Simon A. Wegener[a], Christoph G. Lindenmeir[a], Lixing Li[a], Thomas Baier[a], Matthias Schwartzkopf[c], Sarathlal Koyiloth Vayalil[c,d], Kun Sun[a,b], Peter Müller-Buschbaum[a*]

a. Technical University of Munich, TUM School of Natural Sciences, Department of Physics, Chair for Functional Materials, James-Franck-Str. 1, 85748 Garching, Germany.

b. Helmholtz-Zentrum Berlin für Materialien und Energie GmbH, Hahn-Meitner-Platz 1, 14109 Berlin, Germany.

c. Deutsches Elektronen-Synchrotron DESY, Notkestr. 85, 22607, Hamburg, Germany.

d. Applied Sciences Cluster, University of Petroleum and Energy Studies UPES, Dehradun, Uttarakhand, 248007, India.

*Corresponding author: muellerb@ph.tum.de

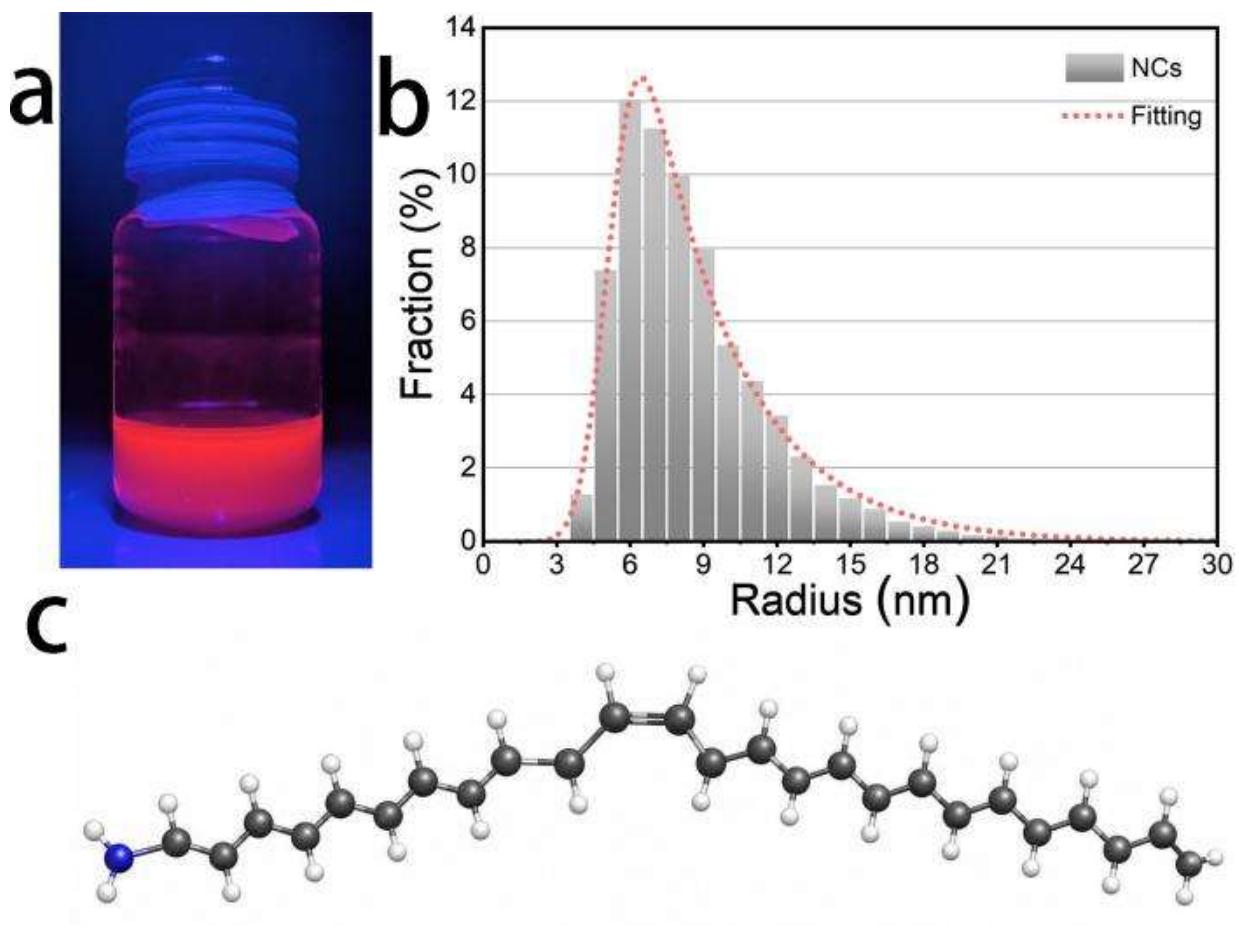

Figure S1. (a) Photograph showing the photoluminescence properties of $CsPbI_3$ perovskite nanocrystals. (b) $CsPbI_3$ perovskite nanocrystal size distribution determined by DLS. (c) Molecular structure of the oleylamine ligand on $CsPbI_3$ perovskite nanocrystals.

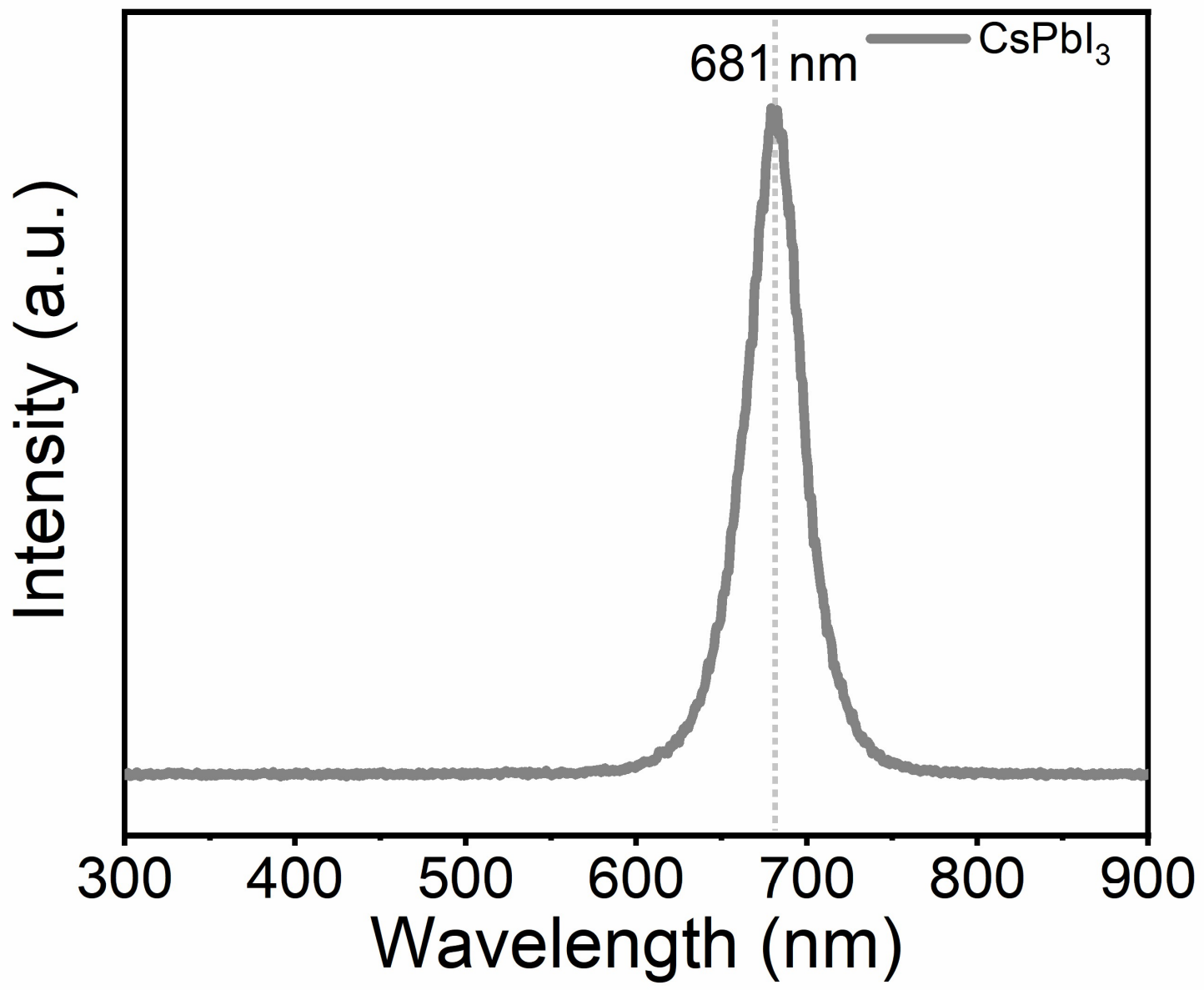

Figure S2. PL spectrum of $CsPbI_3$ perovskite nanocrystals with a maximum at 681 nm.

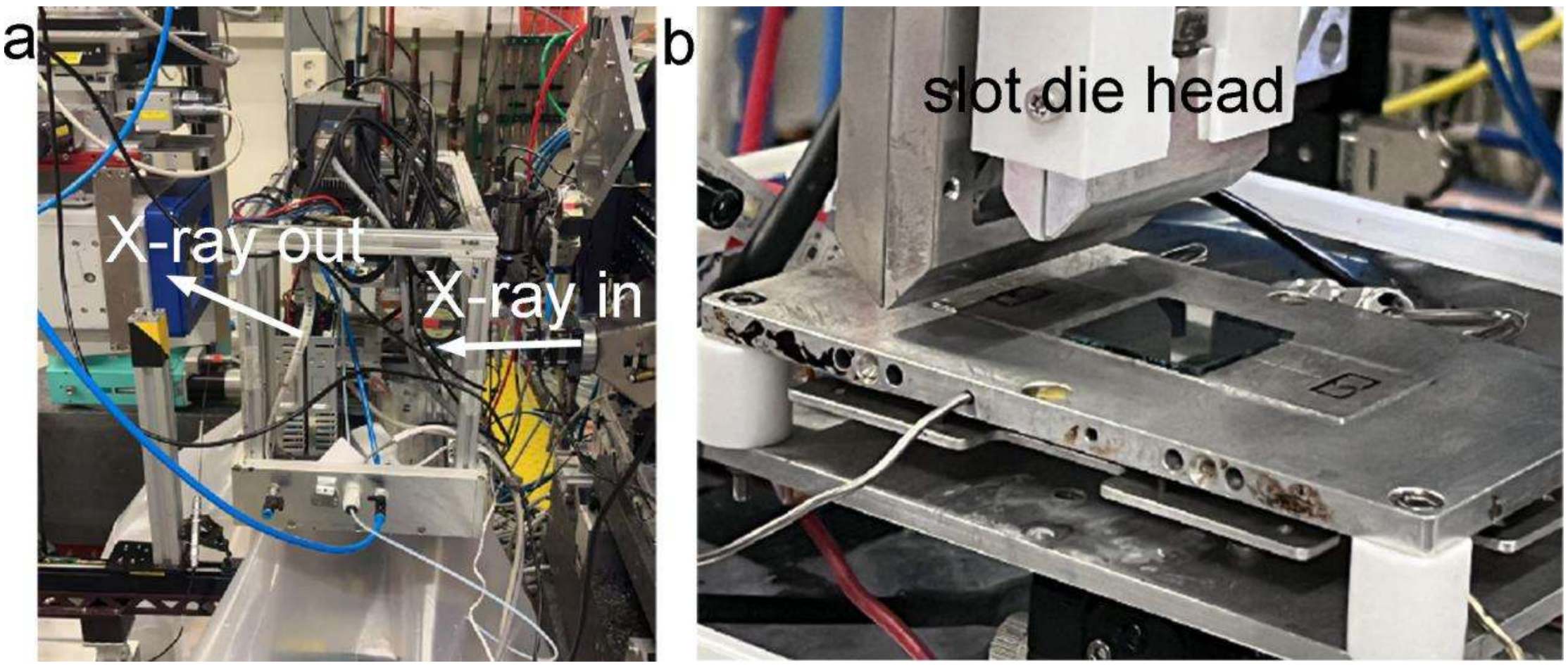

Figure S3. (a) Slot-die coater setup mounted at beamline P03, DESY, for the *in-situ* scattering experiments, and (b) zoomed-in image of the slot-die setup showing the sample table and the slot die head.

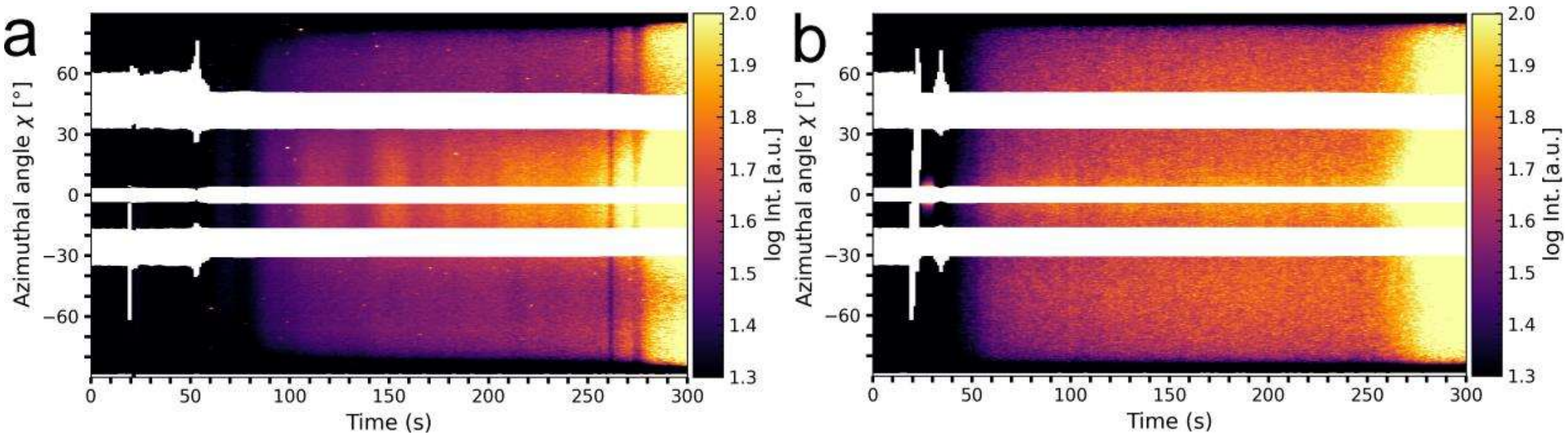

Figure S4. Mapping of the azimuthal cuts along the {100} diffraction peak for (a) control and (b) seeded films during *in situ* printing, highlighting the preferential (001) crystallographic orientation. The white areas indicate the detector gaps.

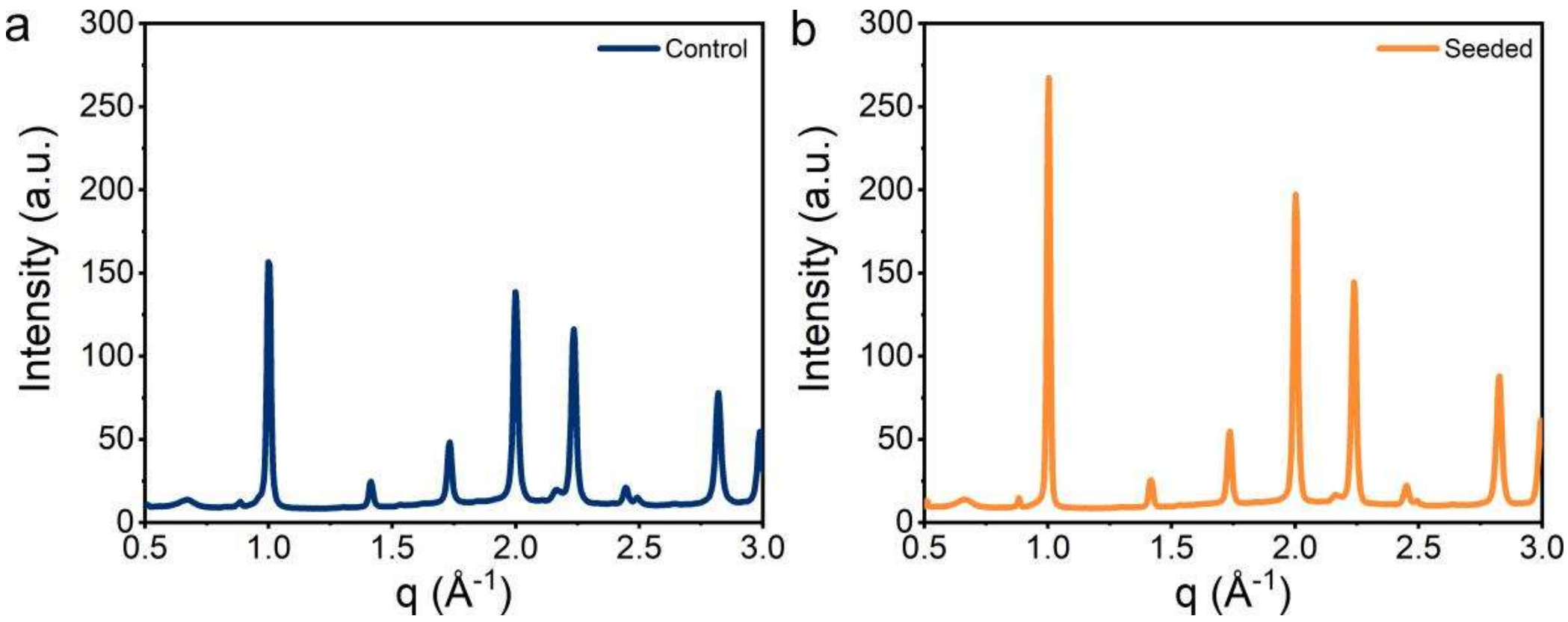

Figure S5. Pseudo XRD patterns extracted from 2D GIWAXS data of the (a) control and (b) seeded samples prepared by slot-die coating.

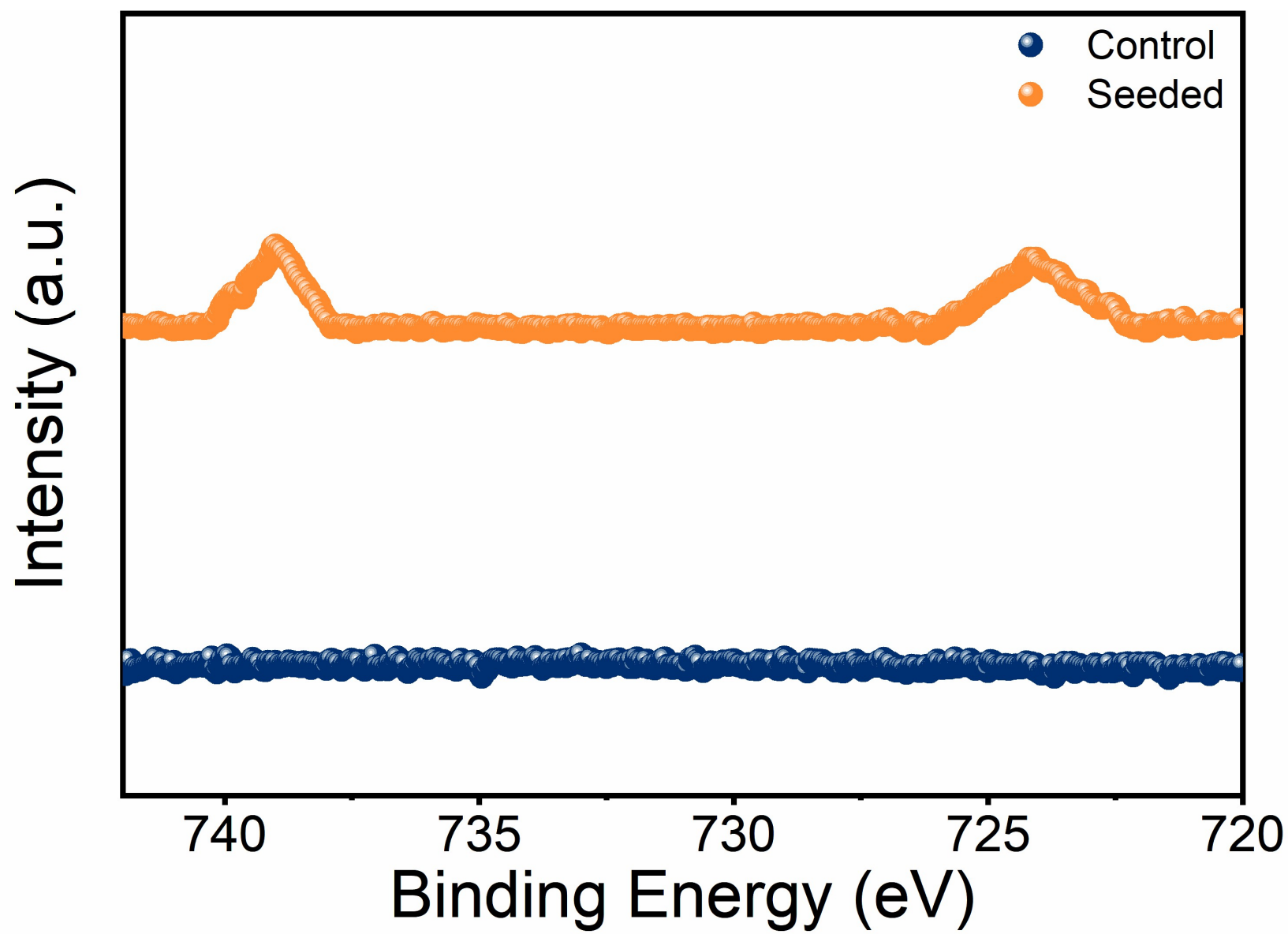

Figure S6. XPS high-resolution spectrum of (a) control and (b) seeded perovskite films.

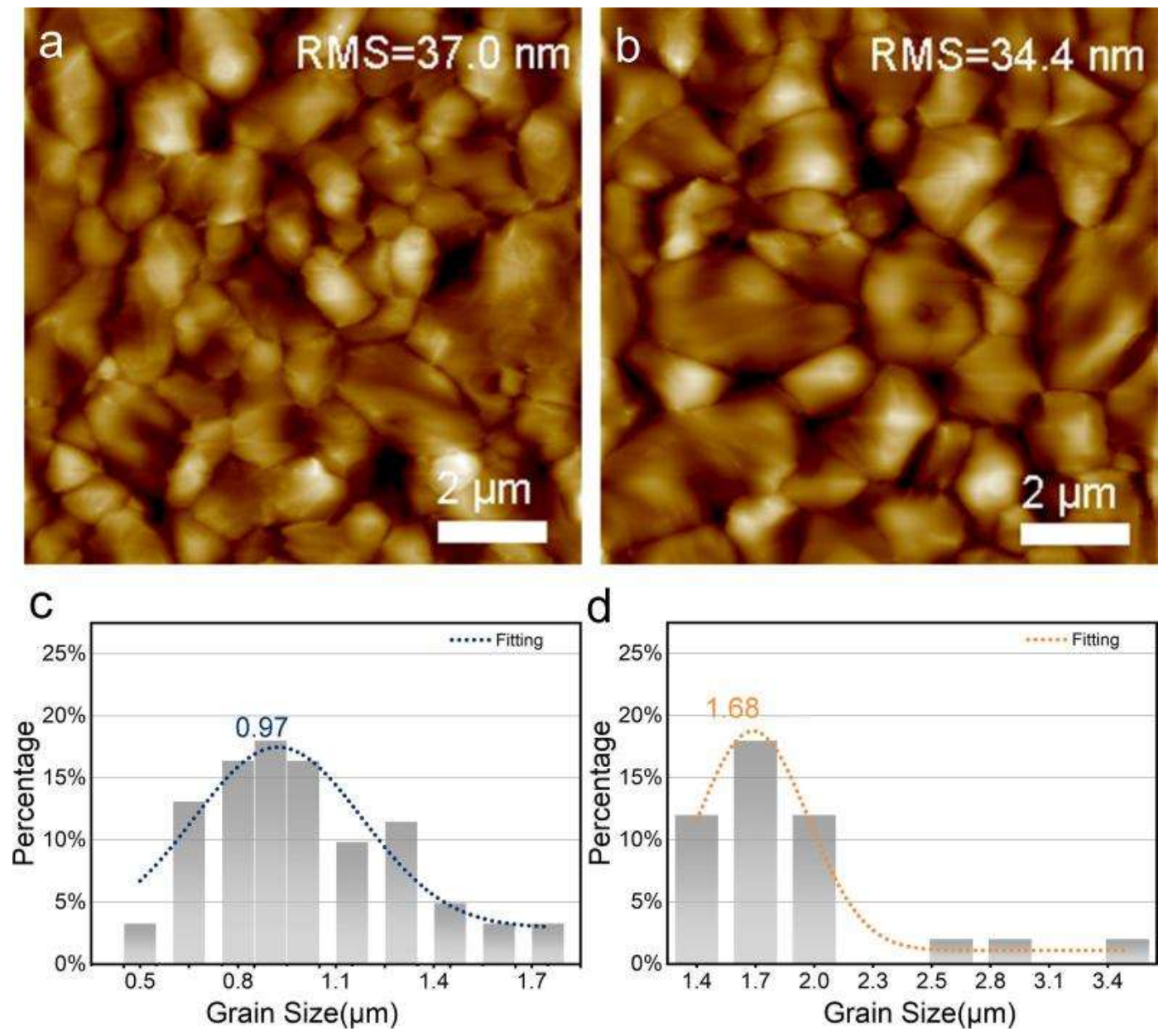

Figure S7. AFM images of (a) control and (b) seeded perovskite films. The statistical grain size distribution of (c) control and (d) seeded perovskite films (n = 50 grains for each sample).

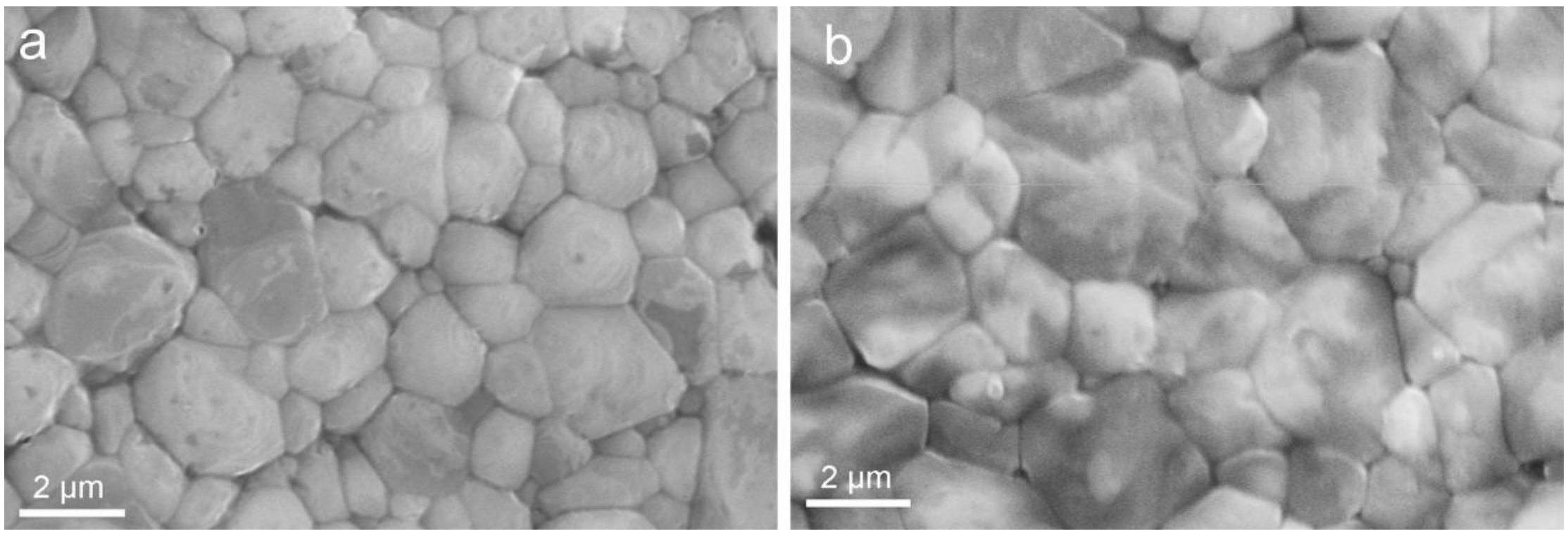

Figure S8. Top-view SEM images of (a) control and (b) seeded perovskite films.

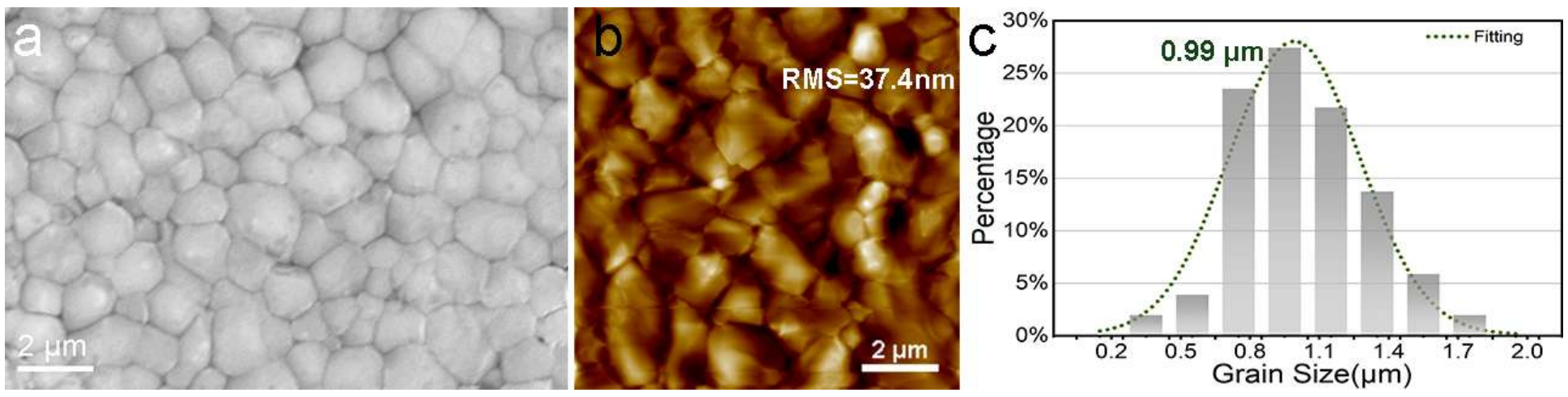

Figure S9. (a) Top-view SEM image, (b) AFM image, and (c) statistical grain size distribution of CsI-only perovskite films.

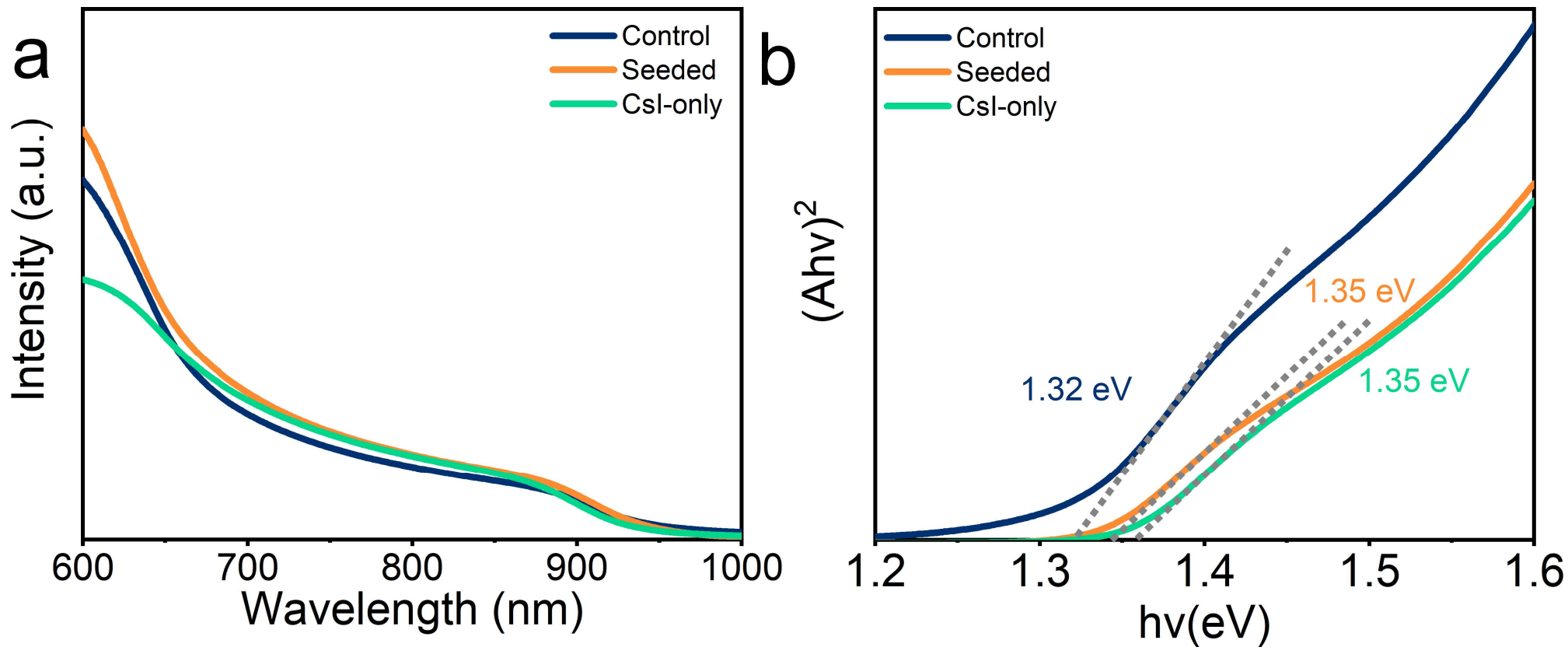

Figure S10. (a) UV-Visible spectra of control CsI-only and seeded films, and (b) their corresponding Tauc plots with the determined band gap energies.

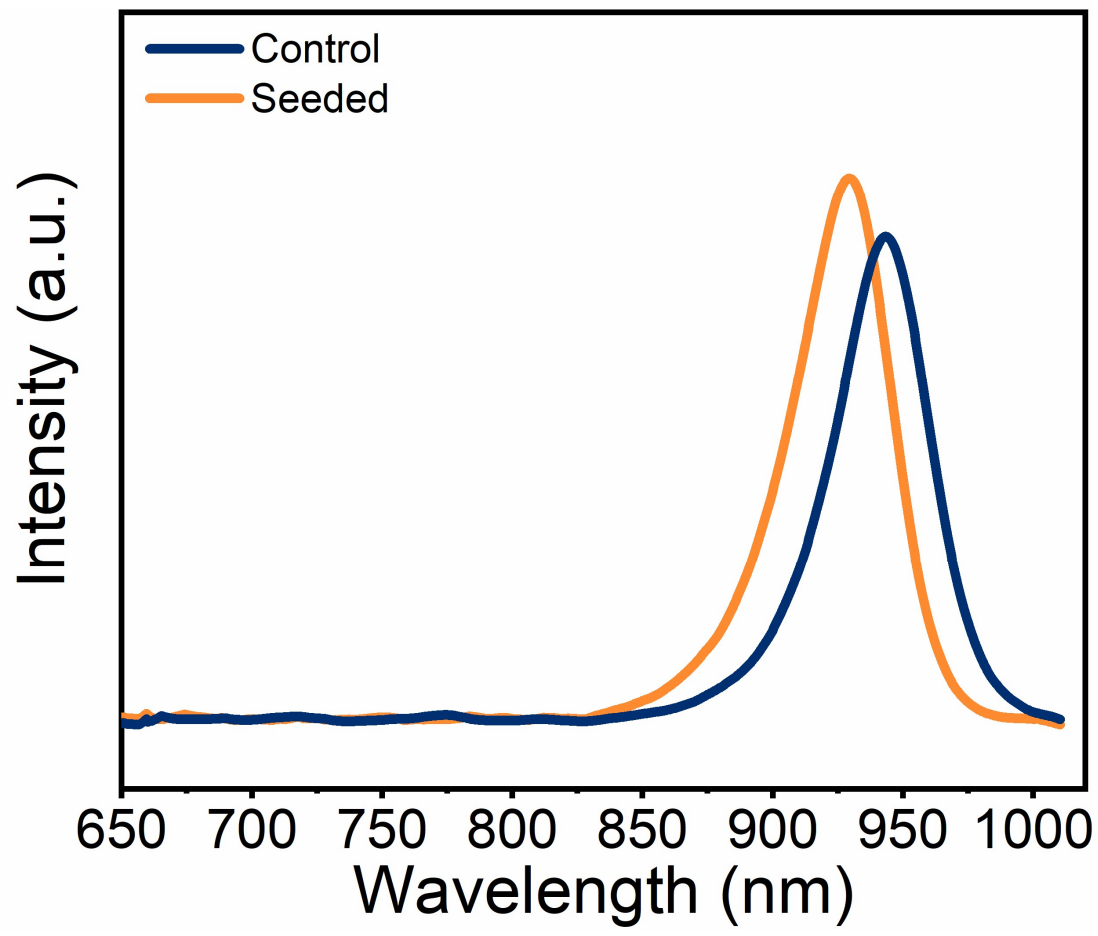

Figure S11. PL spectrum of control (blue) and seeded (orange) perovskite films with a maximum at 943.5 nm and 929.5 nm, respectively.

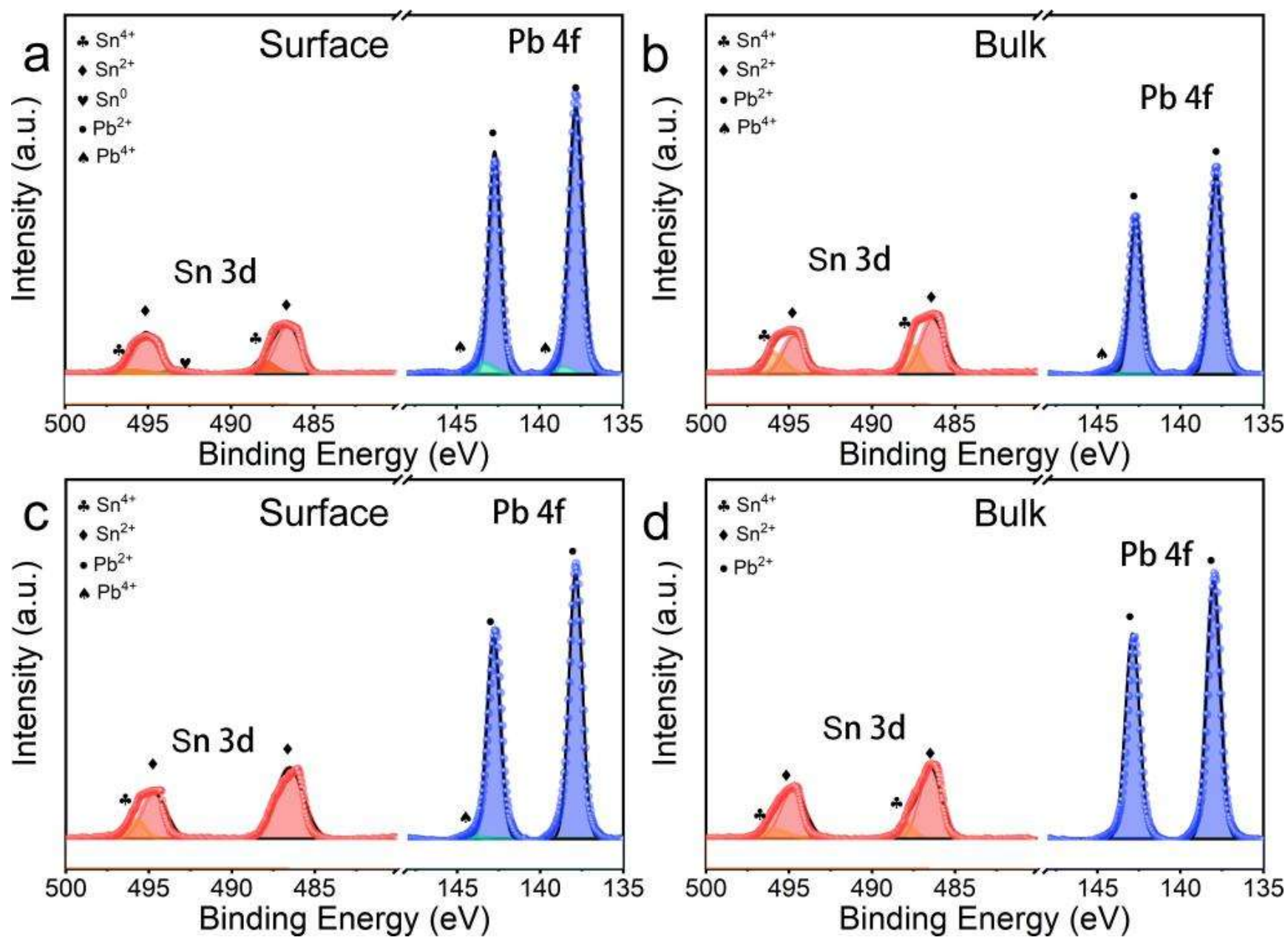

Figure S12. Pb 4f and Sn 3d high-resolution XPS measurements of (a) control sample top surface, (b) control sample bulk region, (c) seeded sample top surface, and (d) seeded sample bulk region.

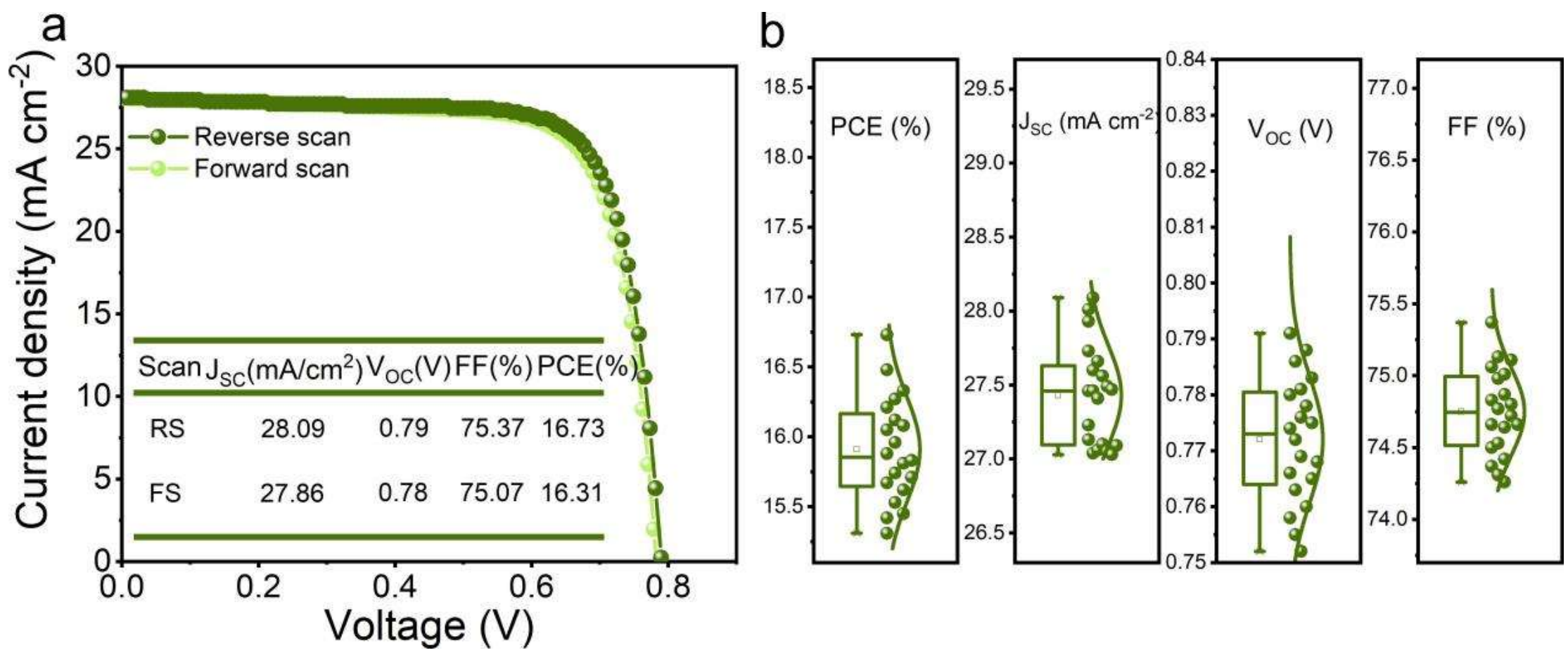

Figure S13. (a) J-V curves with forward and reverse scans of the champion PSCs fabricated from CsI-only perovskite. (b) Box plots of the distribution of PCE, JSC, VOC, and FF values of the PSCs fabricated from CsI-only perovskite (20 devices).

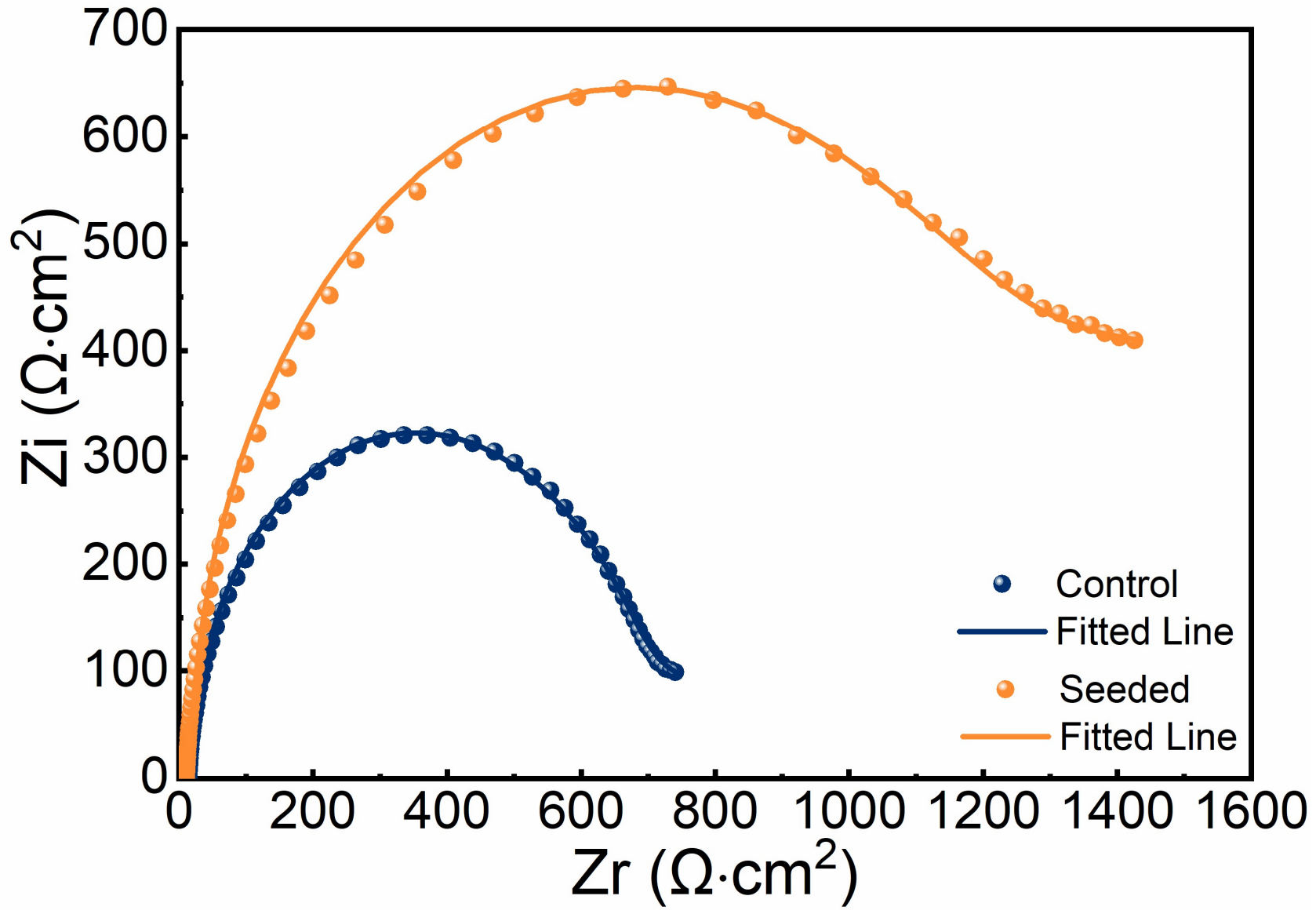

Figure S14. Nyquist plots of electrochemical impedance spectroscopy (EIS) data of the PSCs fabricated from printed control (blue) and seeded (orange) perovskite films.

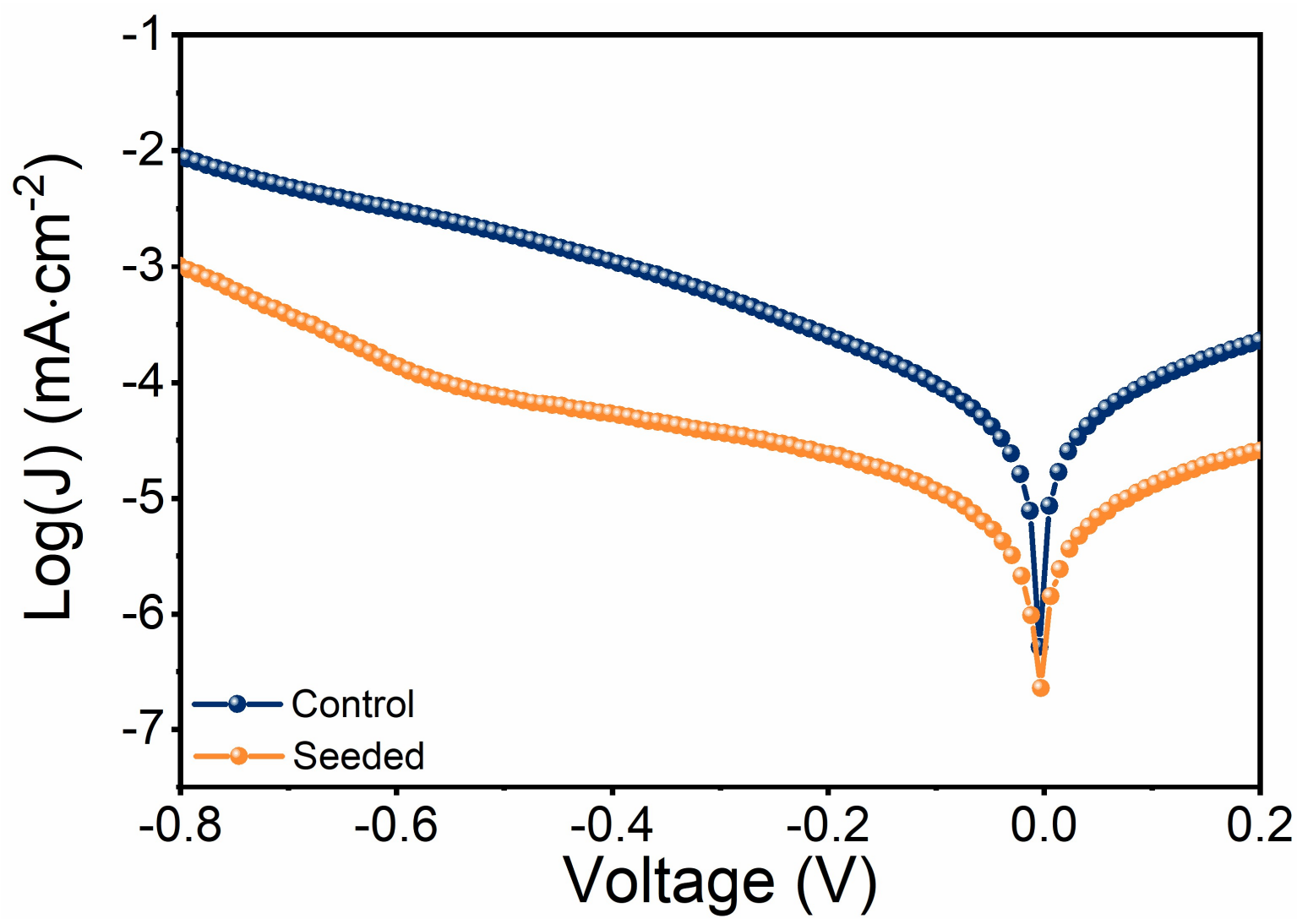

Figure S15. Dark J-V curves of the PSCs fabricated from printed control (blue) and seeded (orange) perovskite films.

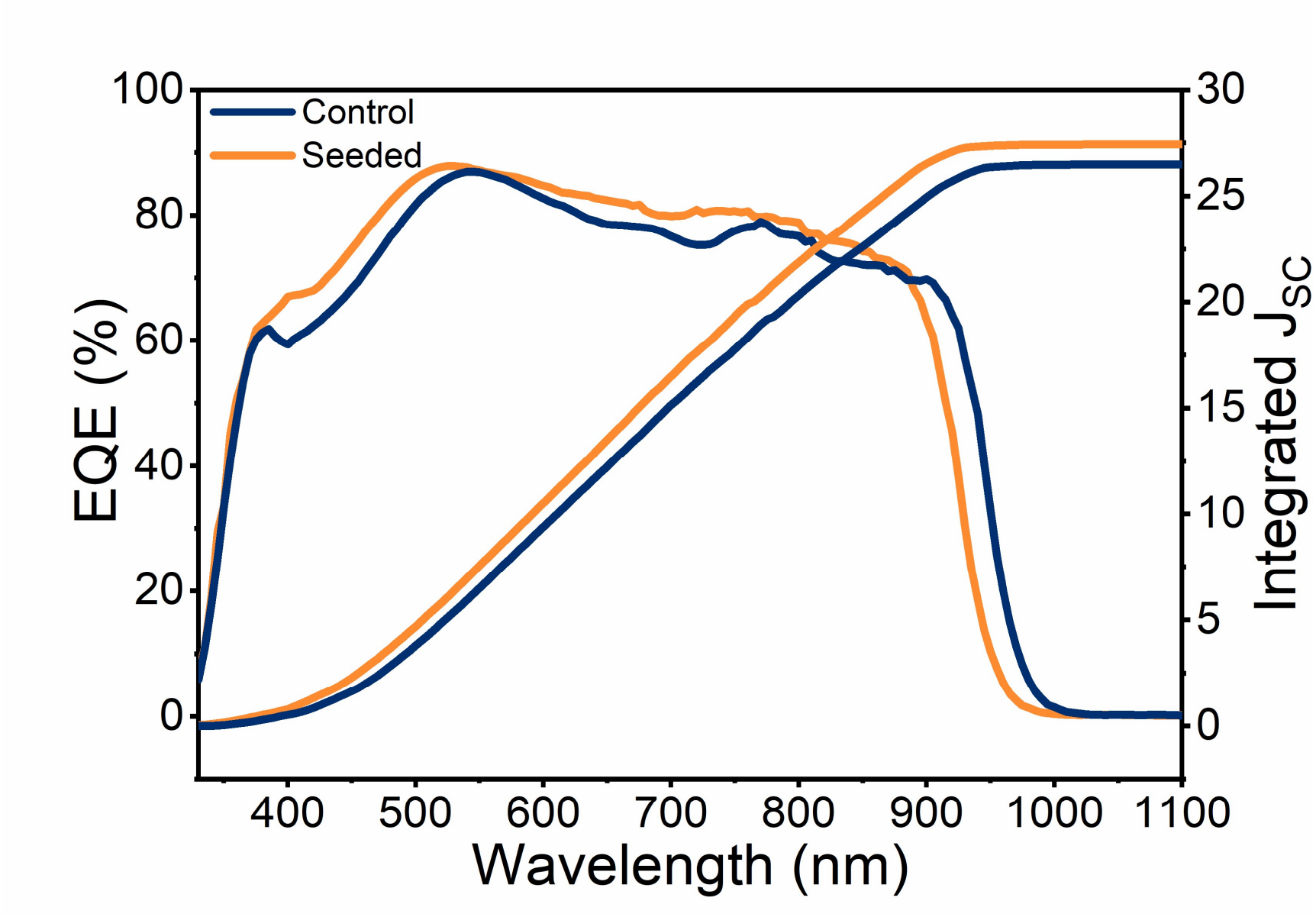

Figure S16. EQE spectra and integrated J_{SC} for the control and seeded PSCs, respectively. Their integrated J_{SC} is 26.63 and 27.58 mA cm^{-2}, respectively.

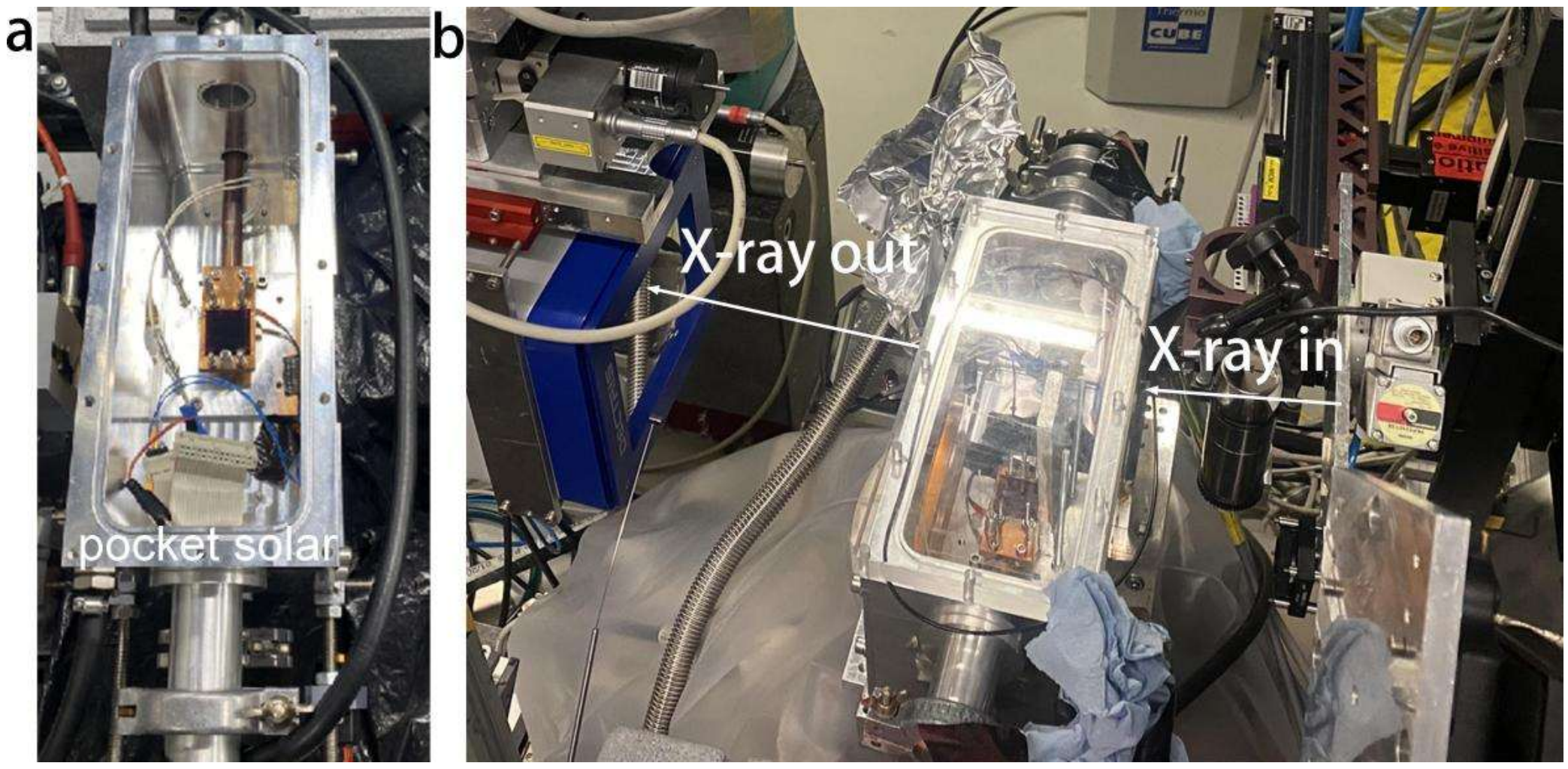

Figure S17. Experimental setup for the *operando* GIWAXS measurements at beamline P03, DESY.

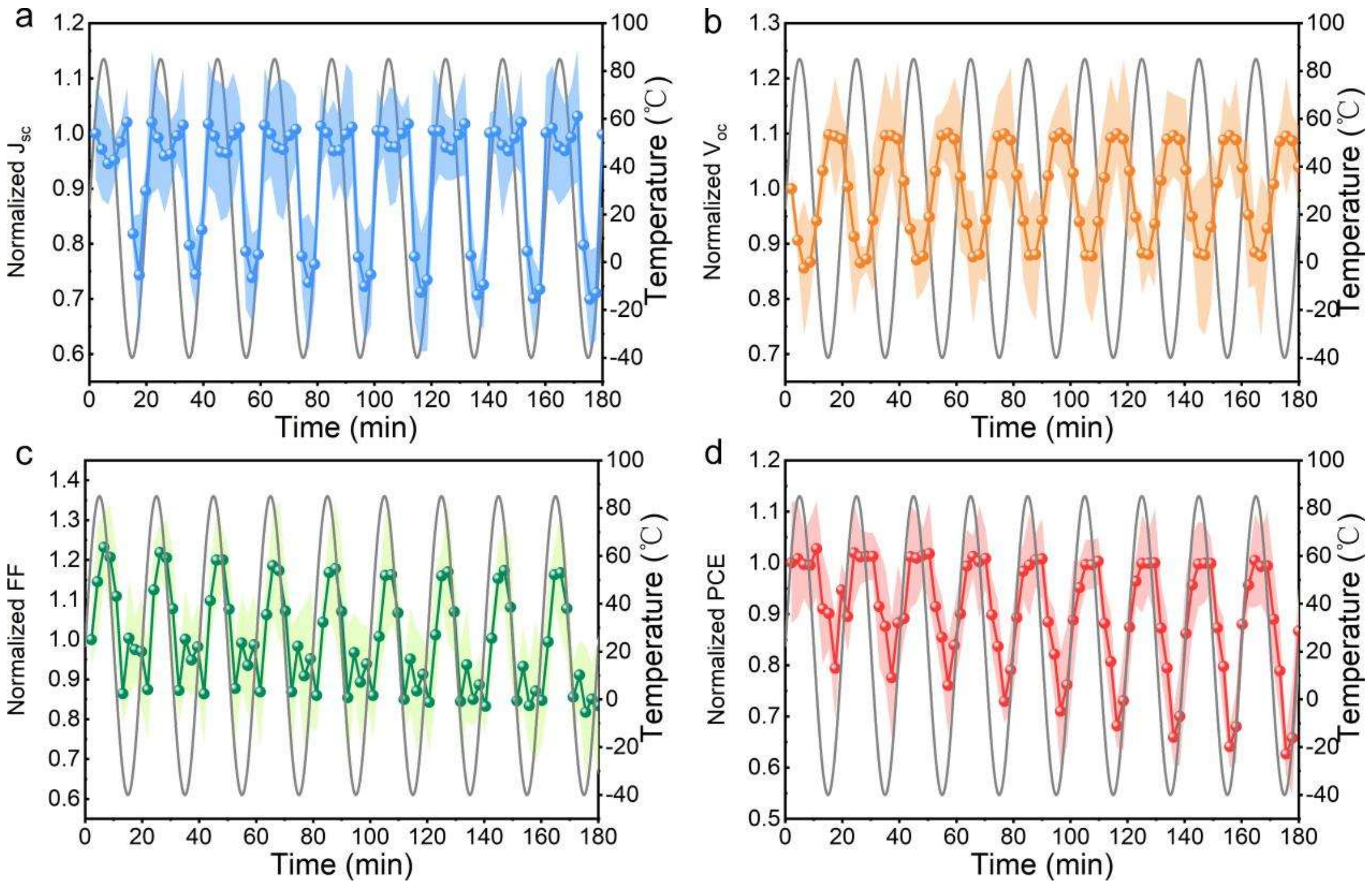

Figure S18. Evolution of the device performance (a) J_{SC}, (b) V_{OC}, (c) FF, and (d) PCE under ISOS-T-3 conditions for PSCs fabricated from CsI-only perovskite films. The solid curves represent the mean values, and the shaded regions represent the error bar.

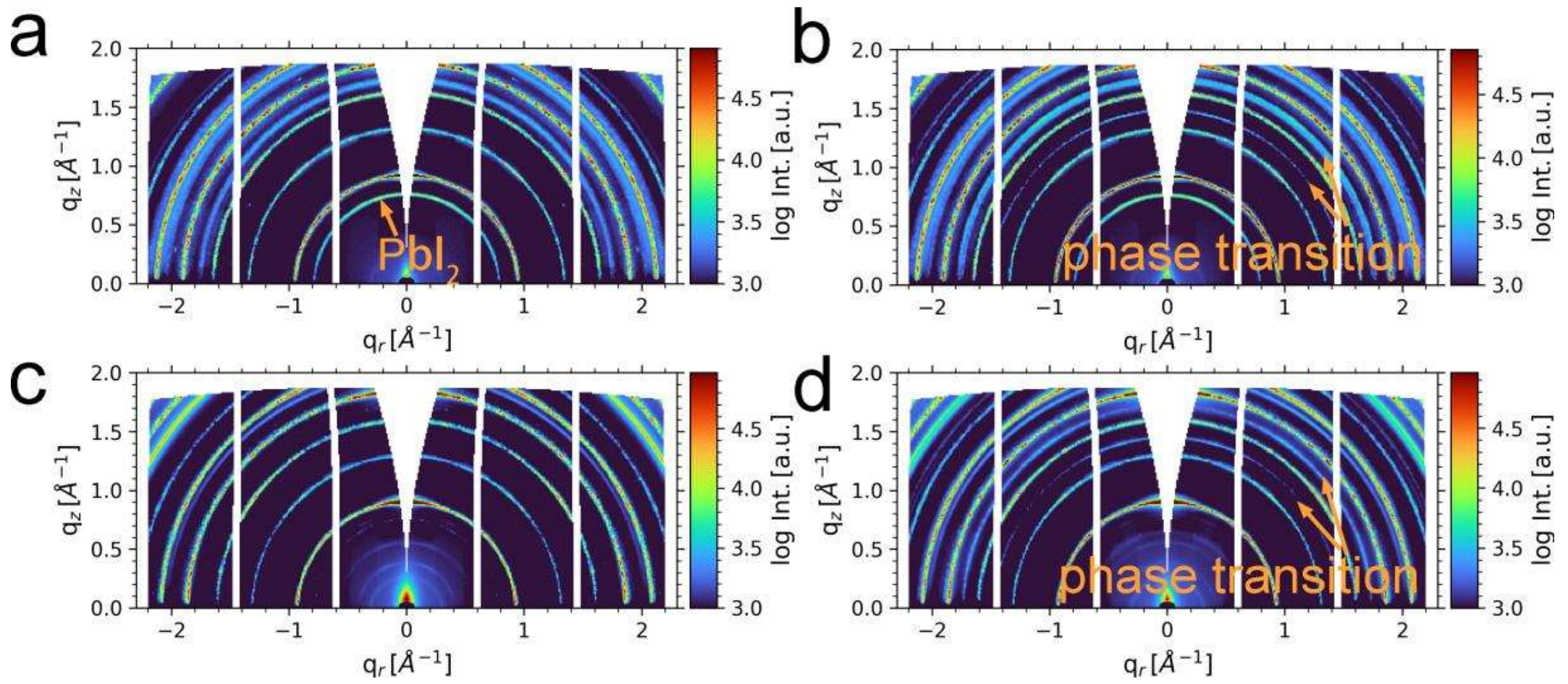

Figure S19. Selected reshaped 2D GIWAXS data of the PSCs fabricated from printed control perovskite films within one thermal cycle at (a) 25 ℃ and (b) at -40 ℃. Selected reshaped 2D GIWAXS data of the PSCs fabricated from printed seeded perovskite films within one thermal cycle at (c) 25 ℃ and (d) at -40 ℃.

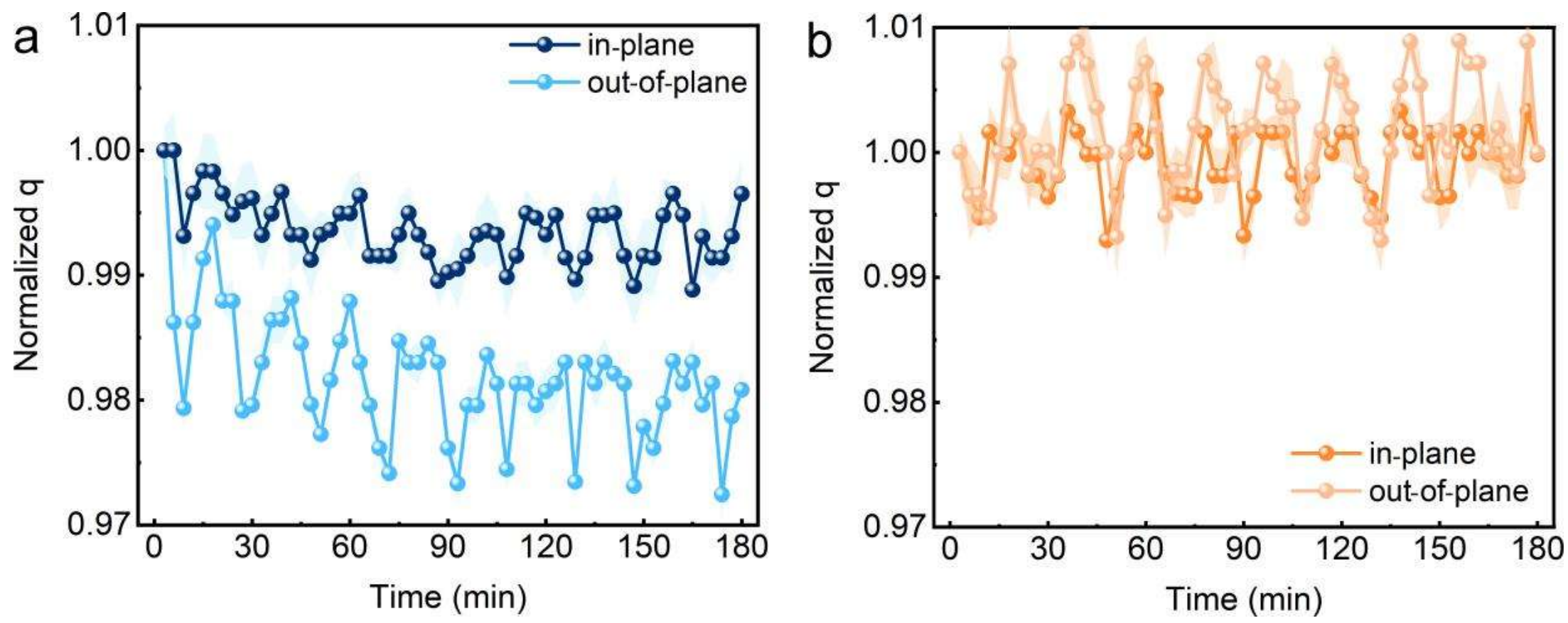

Figure S20. Normalized time-dependent variations in q positions of the (001) diffraction peak of PSCs fabricated from printed (a) control and (b) seeded perovskite films within thermal cycling.

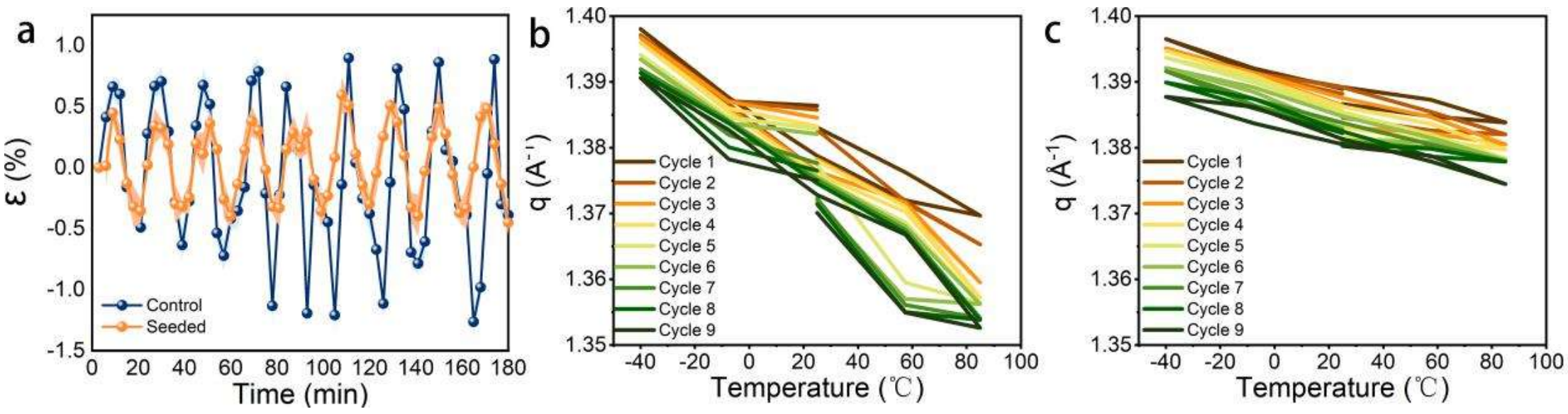

Figure S21. (a) Time-dependent variations in lattice strain of control and seeded samples. Temperature-dependent variations in q positions of the (011) diffraction peak of (b) control and (c) seeded perovskite films within different thermal cycles.

Table S1. Comparison of the photovoltaic performance and stability of recently reported scalable NBG Sn-Pb perovskite solar cells.

Ref.	Year	Deposition method	PCE (%)	Stability	Encapsulation
[1]	2023	High-speed blade coating	16.5	92.8% of the initial PCE retained after 28 days under continuous illumination	Yes
[2]	2024	Blade coating with N_2 gas quenching	18.3	Stability of the scalable blade-coated PSC was not reported	-
[3]	2024	Blade coating with N_2 gas quenching	20.0	Long-term stability of the scalable single-junction PSC was not reported	-
[4]	2025	Blade coating with vacuum-assisted annealing	22.0	Encapsulated NBG mini-module retained 85% of the initial PCE after >1000 h under continuous AM1.5G illumination in ambient conditions (30–40% RH)**	Yes
[5]	2026	Blade coating	21.9	85% of the initial PCE retained after 700 h at 85 °C in an N_2 atmosphere	No
This work	2026	Slot-die coating	18.06	80% of the initial PCE retained after 9 ISOS-T-3 thermal cycles (-40 to 85 °C)	No

Microstrain quantification was performed using the Williamson-Hall method through analysis of GIWAXS diffraction patterns[6]. The total observed peak broadening (β) originates from two distinct contributions: crystallite size effects (β_D) and lattice microstrain (β_ε), as expressed by:

$$\beta = \beta_D + \beta_\varepsilon \quad (1)$$

The crystallite size contribution is determined via Scherrer's equation:

$$\beta_D = \frac{K\lambda}{D\cos\theta} \quad (2)$$

where K = 0.89 represents the dimensionless shape factor, λ denotes the X-ray wavelength, D corresponds to the crystallite dimension, and θ is the Bragg diffraction angle.

The microstrain-induced broadening component follows the relationship:

$$\beta_\varepsilon = 4\varepsilon\tan\theta \quad (3)$$

where ε represents the lattice microstrain magnitude.

By combining Equations (1)-(3), we derive the unified Williamson-Hall relationship:

$$\beta = \frac{K\lambda}{D\cos\theta} + 4\varepsilon\tan\theta \quad (4)$$

This linearized form enables simultaneous determination of crystallite size and microstrain through:

$$\beta\cos\theta = \frac{K\lambda}{D} + (4\sin\theta)\varepsilon \quad (5)$$

The microstrain (ε) is subsequently extracted from the slope of the linear regression between (βcosθ)/λ and 4sinθ/λ, while the y-intercept provides the crystallite size (D) through the Scherrer constant K.

References

[1] Y. Shi, J. Sun, J. Zhou, et al., "High-Speed Deposition of Large-Area Narrow-Bandgap Perovskite Films for All-Perovskite Tandem Solar Mini-Modules," *Advanced Functional Materials* 33 (2023): 2307209.

[2] J. K. Pious, H. Lai, J. Hu, et al., "In Situ Buried Interface Engineering towards Printable Pb–Sn Perovskite Solar Cells," *ACS Applied Materials & Interfaces* 16 (2024): 39399–39407.

[3] S. Siegrist, J. K. Pious, H. Lai, et al., "Stabilizing Solution–Substrate Interaction of Perovskite Ink on PEDOT:PSS for Scalable Blade Coated Narrow Bandgap Perovskite Solar Modules by Gas Quenching," *Solar RRL* 8 (2024): 2400447.

[4] J. Sun, Q. Tian, C. Yu, et al., "Pyridine-Assisted Solvent Engineering for High-Quality Narrow-Bandgap Perovskites in Efficient Tandem Modules," *Nature Communications* 16 (2025): 8997.

[5] H. Gao, J. Wen, S. Zhao, et al., "Crystallization Modulation of Methylammonium-Free Narrow-Bandgap Perovskite for Thermal-Stable All-Perovskite Tandem Solar Modules," *Science Advances* 12 (2026): eadz9089.

[6] R. Guo, D. Han, W. Chen, et al., "Degradation Mechanisms of Perovskite Solar Cells under Vacuum and One Atmosphere of Nitrogen," *Nature Energy* 6 (2021): 977–986.
